# Coherent X‑ray Diffraction Imaging of a Twinned PtRh Catalyst Nanoparticle under Operando Conditions

**DOI:** 10.1021/acsnano.4c15457

**Published:** 2025-06-25

**Authors:** Lydia J. Bachmann, Dmitry Lapkin, Jan-Christian Schober, Daniel Silvan Dolling, Young Yong Kim, Dameli Assalauova, Nastasia Mukharamova, Jagrati Dwivedi, Tobias U. Schulli, Thomas F. Keller, Ivan A. Vartanyants, Andreas Stierle

**Affiliations:** † Centre for X-ray and Nano Science CXNS, 28332 Deutsches Elektronen-Synchrotron DESY, Notkestraße 85, 22607 Hamburg, Germany; ‡ Department of Physics, University of Hamburg, Notkestraße 9-11, 22607 Hamburg, Germany; § Deutsches Elektronen-Synchrotron DESY, Notkestraße 85, 22607 Hamburg, Germany; ∥ 55553 European Synchrotron Radiation Facility (ESRF), 38043 Grenoble Cedex 9, France

**Keywords:** dislocations, single nanoparticle, heterogeneous catalysis, in situ conditions, twin boundary, coherent diffraction imaging, strain

## Abstract

We performed operando Bragg coherent X-ray diffraction imaging under CO oxidation, as well as oxidizing conditions on a precharacterized single PtRh nanoparticle. We found that this (111) oriented particle with truncated octahedral shape is twinned with a Σ3 twin boundary parallel to the SrTiO_3_ (001) support, at the height of the nanoparticle edges. We observed that the average strain at the twin boundary is higher under CO oxidation conditions compared to pure CO or O_2_. In addition, we observed that two new facets were forming during the oxidizing/reducing cycles. Furthermore, we observed mixed edge/screw dislocations at the twin boundary, but only where the {111} side facets meet. Inducing such dislocations changes the local strain and the atomic structure of the nanoparticles, which may create more active sites close to the nanoparticle edges.

## Graphical abstract

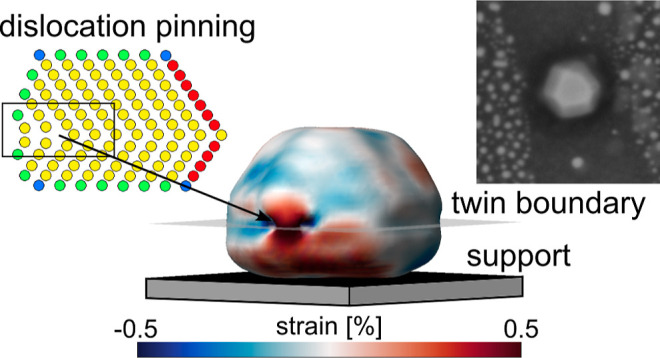

Catalysts play an important role in industrial processes; 80% of the industrially important chemicals worldwide are produced with their assistance.^1^ Also, they play a crucial role in the energy transition, for example, as chemical energy converters.^2^ To develop heterogeneous catalysts with high activity, selectivity, and a longer lifetime, it is crucial to understand structure–activity relations in more details. Therefore, investigating the nanoparticles (NP) strain and shape with high resolution under operando conditions is essential. Investigating a single nanoparticle allows to obtain more detailed information about the shape and strain, beyond the average of a nanoparticle ensemble.^3^

A changing strain field in different gas environments can assist to identify the active sites, since the adsorption of gas molecules can lead to compressive strain.^4,5^ Additionally, modifying the distance between the surface atoms by inducing tensile or compressive lattice strain, can shift the d-band center of the metal, leading to a strengthening or weakening of the bonding to the adsorbed reacting gas molecules,^6^ which directly affects the catalytic activity. The shape defines possible adsorption sites for the gas atoms.^7–9^ It was predicted for truncated octahedral Pt nanoparticles under CO + O_2_ that the edges are the most active sites and that the orientation of the neighboring facets influences the turnover frequency of the edges.^10^ It is obvious, that atoms on surface defects have on average less nearest neighbors than bulk atoms which may make them more active.^11^ New concepts are needed to systematically and purposely increase the defect density or to introduce new specific defects and investigate their role in view of their catalytic activity.

Crystal defects are classified in point, line, planar, and volume defects. Line defects are called dislocations and if the atoms are shifted parallel to the dislocation line, resulting in a spiral ramp of the atom layers perpendicular to the dislocation line, the dislocation is called screw dislocation.^12–14^ In contrast, if the atoms are shifted perpendicular to the dislocation line, then the dislocation is called edge dislocation. Edge dislocations can also be built by inserting or removing an extra half plane of atoms in the lattice. One example of a planar defect is a coherent twin boundary, which is a single atomic plane that separates two neighboring crystal domains each with a specific crystallographic orientation.^15^ For electrocatalysis, it was demonstrated that (multi)­twin boundaries enhance the activity for oxygen reduction reactions^16–18^ and ethanol oxidation reaction.^19^ In gas phase reactions, such as methanol synthesis, an enhanced activity was related to twin boundaries as well.^20^

Under operando conditions, heterogeneous catalysts are exposed to near atmospheric or higher pressures and elevated temperatures, thus representing a dynamical system. Therefore, it is mandatory to investigate the catalyst particles under the operando conditions. Hence, the applied measurement technique needs to allow high resolution shape and strain measurements, while being compatible with these realistic reaction conditions. Bragg coherent X-ray diffraction imaging (BCDI) allows to measure the real space electron density of the crystalline part of a single nanoparticle and the strain distribution in three-dimensions (3D) with a spatial resolution of about 10 nm under these harsh conditions.^3,21–24^ Previous BCDI studies showed that dislocations appear as pipes of missing electron density and analyzing the strain around those pipes allows to determine the type of dislocation.^25,26^

PtRh nanoparticles were reported to exhibit an increased activity for CO oxidation compared to Pt or Rh nanoparticles due to synergistic electronic effects.^27^ Their near surface composition is affecting the activity and depends on the surrounding gases under elevated temperature.^9,22,27^ BCDI results suggest the dealloying of PtRh nanoparticles under O_2_ conditions and the alloying at H_2_ conditions.^28^ This alloying and dealloying behavior was also studied under CO reaction conditions, finding strong indications for a more Pt rich surface composition under reducing conditions and more Rh rich surface under reaction conditions.^22^

As demonstrated by BCDI, the strain, shape, and size of a twinned Pt nanoparticle with a twin boundary with an angle to the substrate can change under CO reaction conditions.^29^ This study, however, lacks the investigation of both parts of the twinned particle, which requires the experimentally challenging recording of several Bragg peaks under operando conditions. It was demonstrated for Au nanoparticles in air, that it is possible to measure Bragg peaks for both parts of a twinned particle.^30^

Here, we report on a BCDI experiment in which we imaged a PtRh nanoparticle with a twin boundary parallel to the substrate and probed both parts of the twinned particle individually. In the plane of the twin boundary, we observed three dislocations, which are pinned at the twin boundary, close to the nanoparticle edges at which {111} type nanoparticle facets meet. Additionally, we followed the strain and shape evolution of this particle under pure Ar, CO + O_2_ + Ar, CO + Ar, and O_2_ + Ar gas mixtures at 430 °C and we found at the twin boundary higher strain under CO oxidizing conditions, than under pure CO or pure O_2_. In the experiment, we observed the formation of two new facets with a high index orientation. Using correlative imaging between in situ BCDI and ex situ pre- as well as postcharacterization by atomic force microscopy (AFM), scanning electron microscopy (SEM), and scanning Auger microscopy (SAM), we obtained additional information about the size, the shape, and the chemical composition of the nanoparticle in the initial and the final state.

## Results and Discussions

### Sample and X-ray Experiment

The sample was prepared by codepositing Pt and Rh on a SrTiO_3_ (STO)(100) support at 850 °C as described in the Methods section, with a nominal Pt content of (58 ± 18)% (for calculation see Supporting Information Section S1). To achieve a shape closer to thermodynamic equilibrium, the sample was postannealed at 1100 °C.

The BCDI experiment was performed at the European Synchrotron Radiation Facility (ESRF, Grenoble, France) at beamline ID01 at a photon energy of 9 keV (λ = 0.138 nm) (see Methods section). The experiment was carried out at 430 °C with a constant total gas flow of 50 mL min^–1^ and a constant pressure of 0.1 bar. The gas composition was switched between pure Ar, reducing conditions (CO + Ar), stoichiometric reaction conditions (4 mL/min CO + 2 mL/min O_2_ + Ar), various overstoichiometric reaction conditions (4 mL/min CO + 3–6 mL/min O_2_ + Ar), and various oxidizing conditions (O_2_ + Ar). The detailed gas conditions can be found in Section S2, together with proof of the catalytic activity of the sample by CO_2_ production.

Before the X-ray experiment, the sample was characterized at the DESY NanoLab^31^ by SEM and AFM, allowing us to obtain information about the size and shape of the nanoparticle. Figure 1 summarizes the microscopy results obtained before and after the experiment. The SEM precharacterization is shown in Figure 1a, the investigated particle has a diameter of (146 ± 3) nm. The hexagonal shape already indicates its 111 orientation. To determine the height before the experiment, line profiles (1) and (3) (see Figure 1e,f) were taken in the AFM image shown in Figure 1c. Subtracting the background from the height value of the top of the particle results in a particle height of (97.3 ± 0.7) nm. To track this particle in the X-ray experiment and to relocate for postcharacterization, Pt markers were formed by ion beam induced deposition (IBID), using a dual beam focused ion beam instrument (see Section S3). In the SEM postcharacterization in Figure 1b, the side facets a-f are more clearly visible than in the precharacterization, since the magnification of the image was increased and a concentric backscattered electron detector was used instead of the secondary electron detector. Comparing the SEM postcharacterization with the precharacterization shows no change of the particle diameter within the error bar of 3 nm. In contrast, comparing the AFM postcharacterization (Figure 1d) with the AFM precharacterization results, the diameter of the particles is seemingly changing. This change appears only in the vertical and not in the horizontal line profile (Figure 1e) and is most likely arising from the convolution of the particle with the shape of different tips for both measurements. The height of the particle slightly increased to (102 ± 5) nm. Note that the error bar has increased, and that the height measured before the experiment still lies within the error bars.

**Figure 1:**
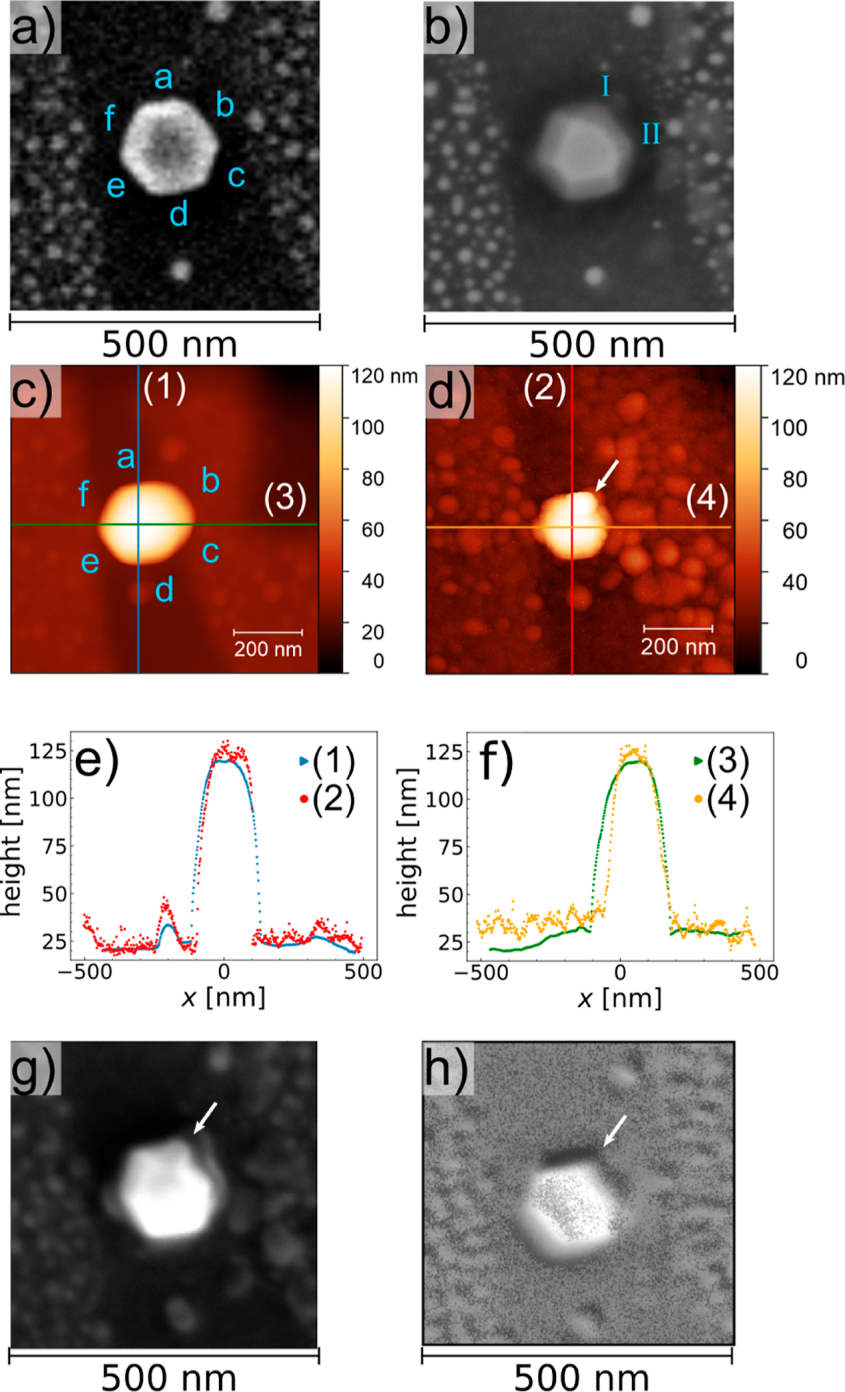
Sample architecture. SEM images (a) before and (b) after the catalysis experiment. For identification purposes, each top side facet is named in (a) by letters in blue, which are kept consistent throughout the whole text. The roman numbers I and II are marking the new facets forming during the catalysis experiment. AFM images (c) before and (d) after the catalysis experiment. (e,f) Comparison of the line profiles taken as indicated by the lines in (c,d). (g) Secondary electron image taken in the SAM instrument after the catalysis experiment (SAM-SEM). (h) Oxygen map measured by SAM after the catalysis experiment, the map was processed as described in Section S11. The white arrow is indicating an agglomeration.

### Nanoparticle Shape under Operando Conditions

In our experiment, we measured the intensity distribution in the reciprocal space in 3D around three different Bragg peaks by changing the detector position in the out-of-plane and the in-plane directions, as shown in Section S4. First, the 111 Bragg peak normal to the top facet was probed, in this reflection, the full particle is imaged. By using Vegard’s rule, the composition of the nanoparticle was calculated from the Bragg peak position to be (50 ± 1)% Pt (see Section S5), which agrees with the nominal composition of around (58 ± 18)% Pt (see Section S1).

As described in the Methods section, the shape of the crystalline part of the particle can be reconstructed from the measured 3D intensity distribution around the Bragg peak. In the top view of the reconstructed shape of the particle (Figure 2a), the {100} and {111} type side facets are clearly visible. The data set (data set 8, for the data set labeling see Section S2) was acquired under a gas flow of 46 mL min^–1^ Ar, 4 mL min^–1^ O_2_, and 4 mL min^–1^ CO. This is the first gas condition under which we also measured on two other Bragg peaks. By comparing the side facet shape with the facet shape of a Wulff construction for a fcc particle (Figure 2b), the three {111} type facets (a, c, and e) and three {100} type facets (b, d, and f) can be identified.

**Figure 2:**
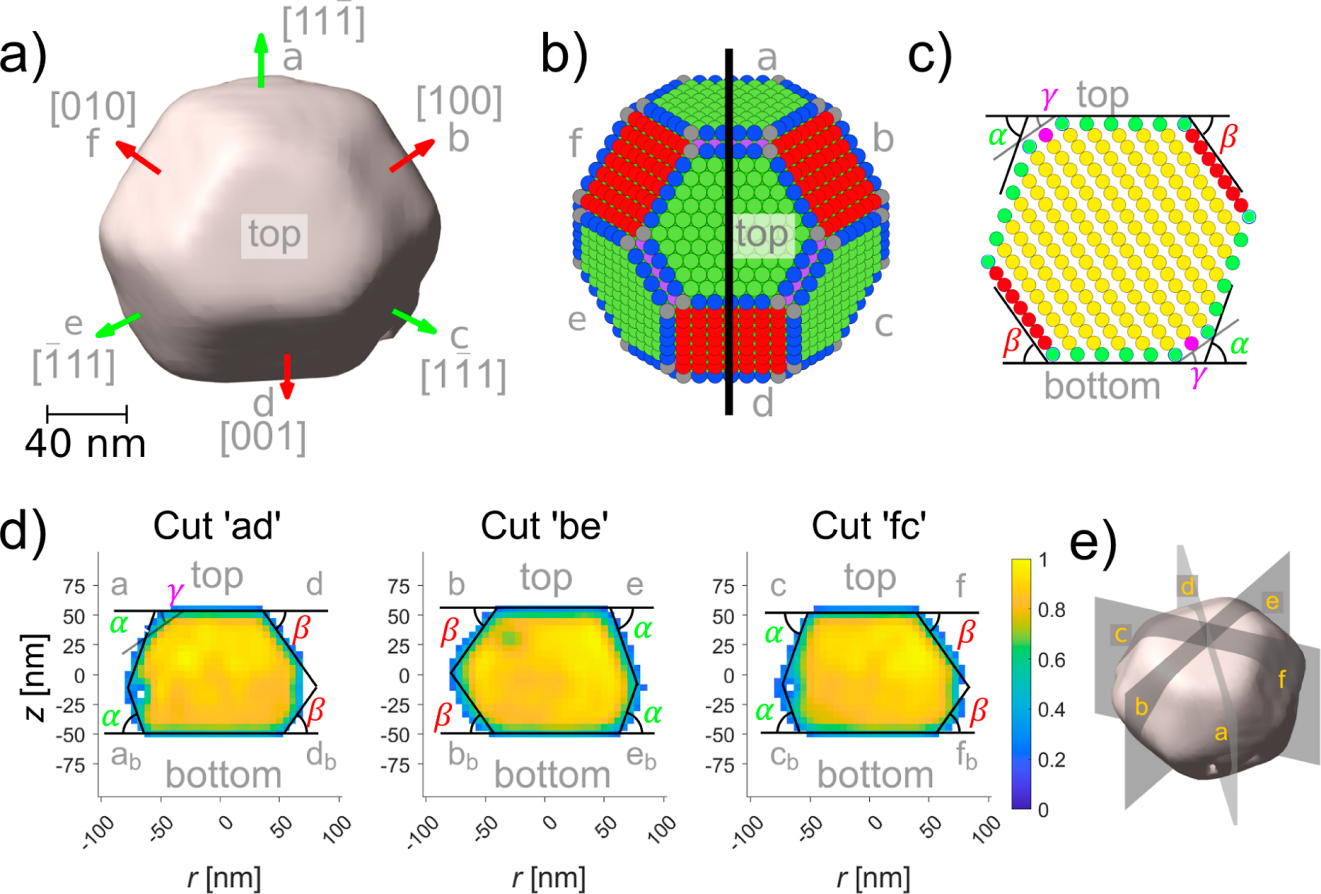
Reconstructed particle shape in comparison with the Wulff construction. (a) Top view on the reconstructed nanoparticle electron density with isosurface value 0.2. The green arrows indicate PtRh {111} type facets and the red arrows the PtRh {001} type facets. (b) Wulff construction of a free fcc(111) particle, {111} type facets are indicated in green, {001} type facets in red, and {110} truncation are indicated in purple. All edge atoms are blue and the corner atoms gray. (c) Cut perpendicular through the Wulff shape as indicated by the black line in (b), with same color coding as in (b). The theoretical angles between the (111) top-/bottom facet and the side facets are shown: α = 70.57° for {111} side facets, β = 54.73° for {100} side facets, and γ = 35.26° for {110} truncation. (d) Cuts perpendicular through the side facets, as indicated in (e). Each bottom side facet is named by the top side facets name with subscript “b”. As a guide to the eye, the theoretical angles between the (111) top-/bottom facet and the side facets are shown for a twinned fcc(111) NP. The colorbar indicates the amplitude. (e) Side view on the nanoparticle electron density with planes perpendicular to the facets.

The Wulff construction fulfills the condition for the surface energies γ: γ_111_ < γ_100_ < γ_110_, which applies for all fcc materials.^32^ The ratio of the surface energies γ_100_/γ_111_ = 1.1, was selected to mimic the shape of the top facet and is in good agreement with experimentally observed ratio for Rh^33^ γ_100_/γ_111_ = 1.2 and the calculated ratio for Rh and Pt of γ_100_/γ_111_ = 1.13–1.19.^34,35^ Additionally, the condition γ_110_/γ_111_ = 1.16 was applied to introduce the observed {110} truncation. The theoretical angle α = 70.57° between the {111} type side facets and the (111) top facet and the angle β = 54.73° between the {100} type side facets and the (111) top facet are drawn in the cut through this model in Figure 2c. Comparing these theoretical angles with the angles in the cuts through the reconstructed shape in Figure 2d (cuts are defined in Figure 2e) confirms the orientation of the top side facets. These cuts also show a small {110} type truncation between some of the {111} side facets and the {111} top surface, with an angle of γ = 35.36°. This is expected for fcc nanoparticles with $\gamma_{110}/\gamma_{111}<\sqrt{\left(3/2\right)}$ .^32^

In contrast to the side facets of the top half, the orientation of the side facets of the bottom half (a_b_–f_b_) do not agree with the prediction from the Wulff construction (Figure 2c). By comparing the theoretical angles with the measured angles between the bottom facet and the bottom side facets, we found that instead of a {111} side facet below a {100} side facet and vice versa, all {111} facets have a {111} facet below, and all {100} facets have a {100} facet below.

Inspecting the reconstruction from the asymmetric (020) Bragg peak at 46 mL min^–1^ Ar, 4 mL min^–1^ O_2_, and 4 mL min^–1^ CO shows only the reconstructed shape of the top part of the particle (see Figure 3a). This means that the (020) Bragg peak belongs only to the lattice of the top part of the particle, thus evidencing the nanoparticle twinning, since the asymmetric Bragg peaks are sensitive to the stacking of the nanoparticle layers. To investigate the bottom part of the nanoparticle, we searched for an asymmetric Bragg peak with a rotation of 180° to the asymmetric Bragg peaks of the top part. Indeed, we found and measured the rotated (220) Bragg peak, whose reconstructed shape agrees with the bottom half of the particle. Thus, the particle is twinned with a twin boundary parallel to the substrate, and the bottom part is rotated by 180° with respect to the top part, which is characteristic for a coherent Σ3 twin boundary. Each facet of the top part has therefore a facet with the same orientation as the neighboring facet of the bottom part, as observed in Figure 2d and modeled in Figure 3b. This model is constructed by cutting the Wulff shape of an fcc(111) particle and rotating the bottom part by 180°. Additionally, the truncation of the nanoparticle induced by the substrate–nanoparticle interface is considered, and for clarity {110} truncations like in Figure 2 are neglected. The cut through this model in Figure 3c shows the change of the stacking sequence from ABC to CBA at the twin boundary, and the angles between the bottom/top facet and the side facets are in very good agreement with the experimental results.

**Figure 3:**
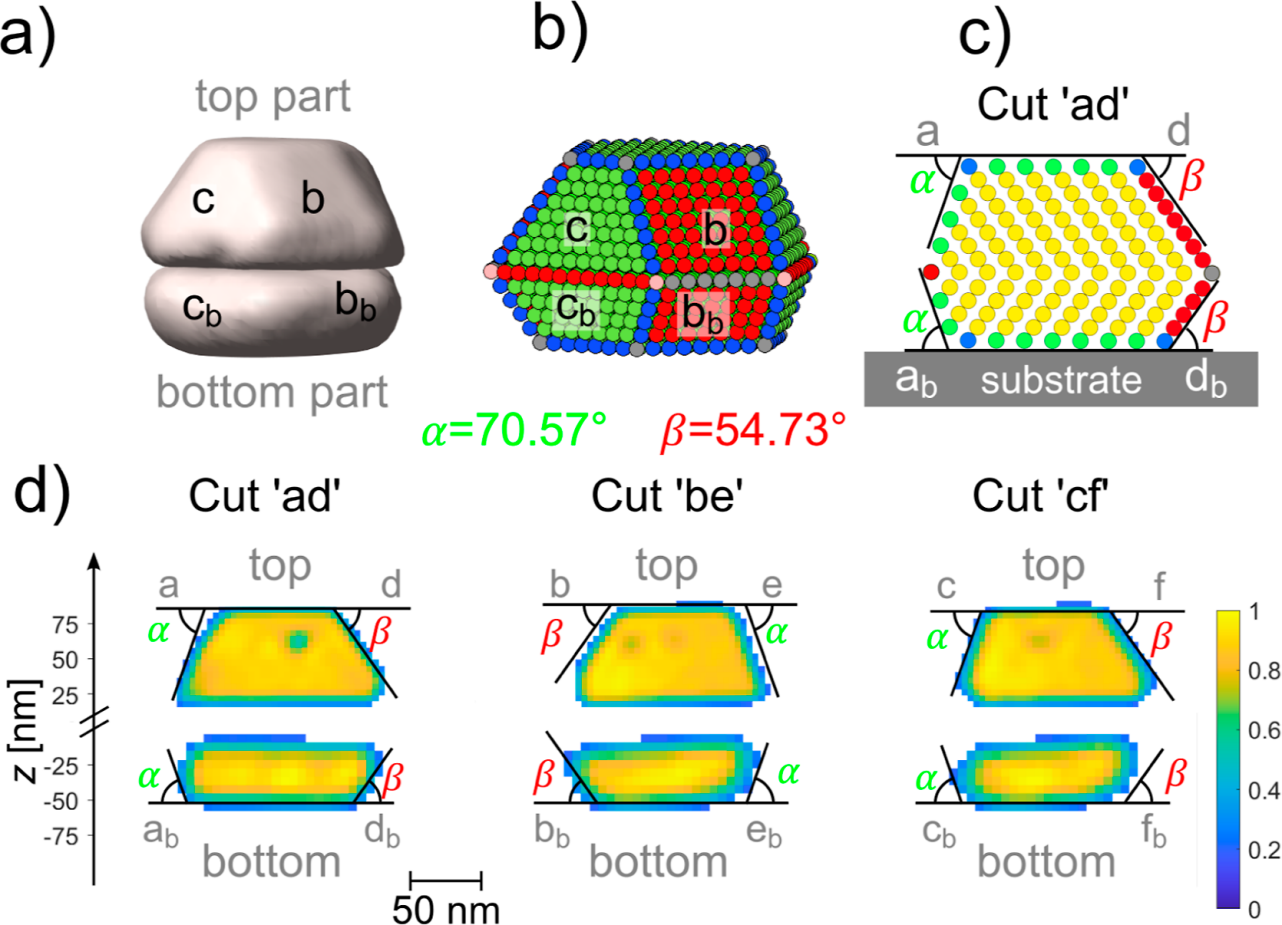
Reconstructed shape of the top part and the bottom part in comparison with an atomistic model. (a) Reconstructed image of two data sets collected at the 020 reflection (top part) and the 220 reflection (bottom part) with isosurface value 0.2. Both data sets (data sets 9 and 10, as labeled in Section S6) were acquired at the same gas condition as Figure 2. (b) Model of an fcc particle with a Σ3 twin boundary parallel to the substrate surface, including the truncation of the nanoparticle due to the substrate, resulting in a smaller bottom than top part. {111} type facets in green, {001} type facets are in red. For clarity {110} truncations like in Figure 2 are neglected. (c) Cut through the atomic model, perpendicular through the side facets f and c. The theoretical angles between the {111} top-/bottom facets and the side facets (α = 70.57° for {111} side facets and β = 54.73° for {100} side facets) are shown. (d) Cuts through the reconstructed amplitude of the asymmetric Bragg reflections 020 and 220. The cutting planes are defined in the same way as for Figure 2d. As guide to the eye, the theoretical angles between the {111} top-/bottom facets and the side facets are drawn. Since both parts are reconstructed independently, the gap between both parts is arbitrary and does not correspond to an actual physical gap between the parts.

The sum of the heights in the cuts through the reconstruction of both parts of the particle (see Figure 3d) show together the same height within the precision of the measurement, as the cuts through the reconstruction from the 111 reflection at the same gas condition. The height can be calculated more precisely from the 3D intensity distribution around the Bragg peak in reciprocal space, as described in Section S6. The height from the bottom part individually (220 reflection) plus the height of the top part individually (020 reflection) is (107 ± 4) nm for the first condition at which all three reflections were measured. This corresponds within the error bars to the total height of (105 ± 5) nm, calculated at the same conditions from the 111 reflection, which excludes the possibility of a disordered region or a third twin grain between the top and the bottom part. This total height also agrees well with the height of (102 ± 5) nm measured by AFM after the experiment (see Figure 1e,f).

While twin boundaries with an angle to the substrate are reported for 4d transition metal nanoparticles prepared by various deposition techniques^29,33,36^ and are common in real catalysts,^20^ particles with twin boundaries parallel to the substrate are, to our knowledge, only reported for nanoparticles prepared by dewetting.^30^ We propose that thermal mismatch induced strain during the rapid cooling of the particle after the postannealing may have induced the stacking inversion of the bottom part. Since the lattice misfit between the support and the nanoparticle is at room temperature 0.18% larger than at the postgrowth annealing temperature (see Section S7), an additional shear stress is induced on the bottom of the nanoparticle during cooling down. Such shear stresses are reported to induce twin boundary migrations.^37^ In this scenario, the local displacement associated with the twin boundary migration was moving through the particle up to the edges between the side facets, where it got trapped. A similar twin boundary migration was reported for strain at the interface between gas phase and nanoparticle.^29^

Throughout the experiment, under various gas conditions, the height of the twin boundary above the support did not change, which is in contrast to a previously reported twin boundary migration under reaction conditions.^29^ It was reported that the interface strain between the gas phase and the nanoparticle is driving this migration to minimize facets with a higher interface energy. In our case, however, the ratio between the different facet types (the {111} and {100} type facets) would not change with a twin boundary migration. Thus, the interface energy is identical for all heights of the twin boundary above the support, and therefore, there is no driving force for a twin boundary migration.

### Formation of New Facets

Further on, we observed the formation of two new facets throughout the experiment. They are visible in the reconstructed shape, as shown in Figure 4 and in the SEM images after the experiment (Figure 1b, indicated by roman numbers I and II). Their appearance is more visible in the stereographic projection (Figure 4, right column), which is a perspective projection of the reconstructed 3D image onto a plane. Thus, each spot corresponds to one facet as labeled in the image Figure 4a and the formation of a new facet is visible in the appearance of a new spot (Figure 4b,c). The first new spot was observed under CO + Ar and the second, under O_2_ + Ar. Comparing the averaged measured spot position with the expected position of several facet orientations shows that the new facet I is $\left(42\overset{®}{1}\right)$ oriented and the new facet II is $\left(7\overset{®}{1}2\right)$ oriented. The error bar on the assignment of the vicinal surface orientation is a few degrees; see Section S8. The structure of these high index facets is shown in Section S9. The new facet I has {111} oriented terraces with monatomic {111} + {100} faceted steps, and the new facet II consists of a {100} terrace with a monatomic “zigzag” shaped step ({101} + {111} faceted). Thus, both new facets have more steps, so there is a higher number of undercoordinated surface atoms compared to {111} and {100} surfaces. Hence, the total number of undercoordinated surface atoms increased after the formation of the new facets, leading to more adsorption sites for CO and O_2_ molecules under reaction conditions.^29,34,38^

**Figure 4:**
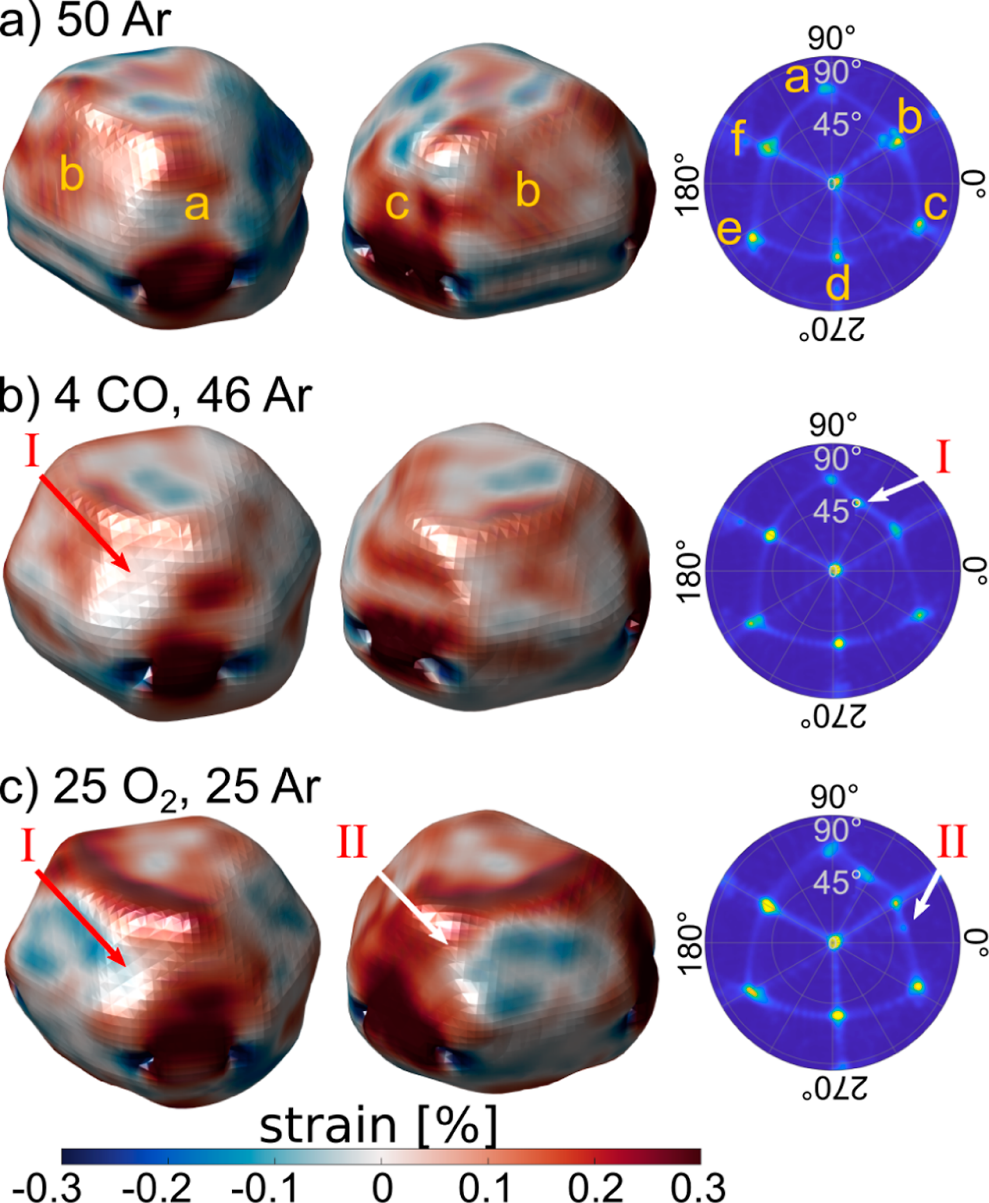
Reconstructed strain and stereographic projections demonstrating the formation of new facets. Two different views of the NP shape with the local strain component ϵ_*zz*_ in the direction of the scattering vector (111) and corresponding stereographic projections. In the stereographic projection correspond the azimuth angle to the azimuth around the nanoparticle and the radius is the angle between the top facet and the top side facets. (a) Initial state (data set 1), (b) first appearance of the $\left(42\overset{®}{1}\right)$ oriented new facet I (data set 7) and (c) first appearance of the second, $\left(7\overset{®}{1}2\right)$ oriented new facet II (data set 15). The gas inlet is given in mL/min.

The aim of this experiment was not only to investigate the evolution of the shape but also the evolution of the strain of the nanoparticle during cycles of oxidizing/reducing/reaction conditions. Strain is a tensor, which can be derived from the atomic displacement field ***u*** (***r***). As described in the Methods section, the displacement field can be obtained from the phase, which is retrieved from an iterative phase retrieval algorithm. One should keep in mind that only the strain component in the direction of the scattering vector of the Bragg peak is quantified. For example, when measuring the 111 reflection, the normal strain component ϵ_*zz*_ with ***z*** = (111) is determined.

Following the evolution of the strain, ϵ_*zz*_ shows no direct relation between the strain and the formation of the new facets (see Section S10). This observation suggests that the formation of new facets is not induced by strain relief. The first new facet was most likely formed under CO + Ar gas conditions. PtRh nanoparticles are reported to be Pt terminated after the growth and under CO conditions.^22^ DFT calculations suggest for pure Pt nanoparticles that there is a thermodynamic driving force at CO saturation to induce more undercoordinated Pt atoms on the surface. This formation of vicinal surfaces was also found experimentally.^29,38,39^ The second new facet was formed under 25 mL/min O_2_ + 25 mL/min Ar. Formation of new high index facets under O_2_ was previously observed for Pd.^40^ Additionally, it was shown for small Rh particles that the formation of oxides under O_2_ can lead to shape changes.^33^ Note that both new facets were formed at the corners, while most studies have focused on the formation of new facets at the edges between the top facet and the side facets. Interestingly, both new facets were formed between the dislocations at facets a and c, which will be discussed below.

At the end of the experiment, the particle was exposed to 50 mL/min pure O_2_ at 430 °C to oxidize the nanoparticle. With increasing the oxygen partial pressure, the high index facets are supposed to vanish in favor of surface or bulk oxide formation.^34,41^ For PtRh nanoparticles, it was reported that Rh is segregating to the surface, since Rh has a higher affinity to oxygen and the Rh surface oxide is more stable and forming more easily than Pt oxide.^28,42–45^ Interestingly, the AFM and SAM-SEM measurements after the experiment (Figure 1d,g) show an agglomeration (indicated by an arrow) at the new facet I, which is oxygen enriched as shown in the Scanning Auger microscopy (SAM) oxygen map in Figure 1h. Since the signal of both Rh and Pt is reduced in this area, this is pointing to Rh or mixed bulk oxide formation. It was not possible to measure BCDI at this final condition, hence there is no information if the two new facets vanished in favor of the oxide formation. The SEM image shown in Figure 1b was measured after the AFM, SAM-SEM and SAM images and after cleaning the sample with 1 × 10^–6^ mbar H_2_ at 300 °C. The direct comparison with the SAM-SEM image taken before the H_2_ treatment shows that the agglomeration vanished due to the treatment. The SEM image shows clearly both vicinal facets I and II. This means that either the vicinal facets are reformed due to the H_2_ reduction treatment or the vicinal facets did not vanish. The latter one is more likely, since the experiments reporting the refaceting toward lower index facets were performed at higher oxygen partial pressures or at higher temperatures.

### Dislocation Related Strain Field Determined from the 111 Bragg Reflection

Figure 5 shows cuts of the local strain component ϵ_*zz*_ in horizontal planes at the twin boundary (Figure 5a), 30 nm above the twin boundary (Figure 5b), and perpendicular to facets a and d (Figure 5c) under various gas conditions. The first important observation in Figure 5a are the gaps parallel to the {111} type facets a, c, and e (the gap parallel to e is more clearly visible in data set 1, see Section S12). Such gaps in the electron density appear if the reconstruction is failing at these points, due to a very high strain induced for example by dislocations. In these cuts, one can also observe an opposite sign in strain at the border of the gap toward the particle center than at the border at the particle surface. This sign change is also visible in the vertical cuts through the strain (Figure 5c), indicating a dislocation. The three dislocations are lying in the twin boundary plane at the conjunction of the {111} facets of the top part and the {111} facets of the bottom part.

**Figure 5:**
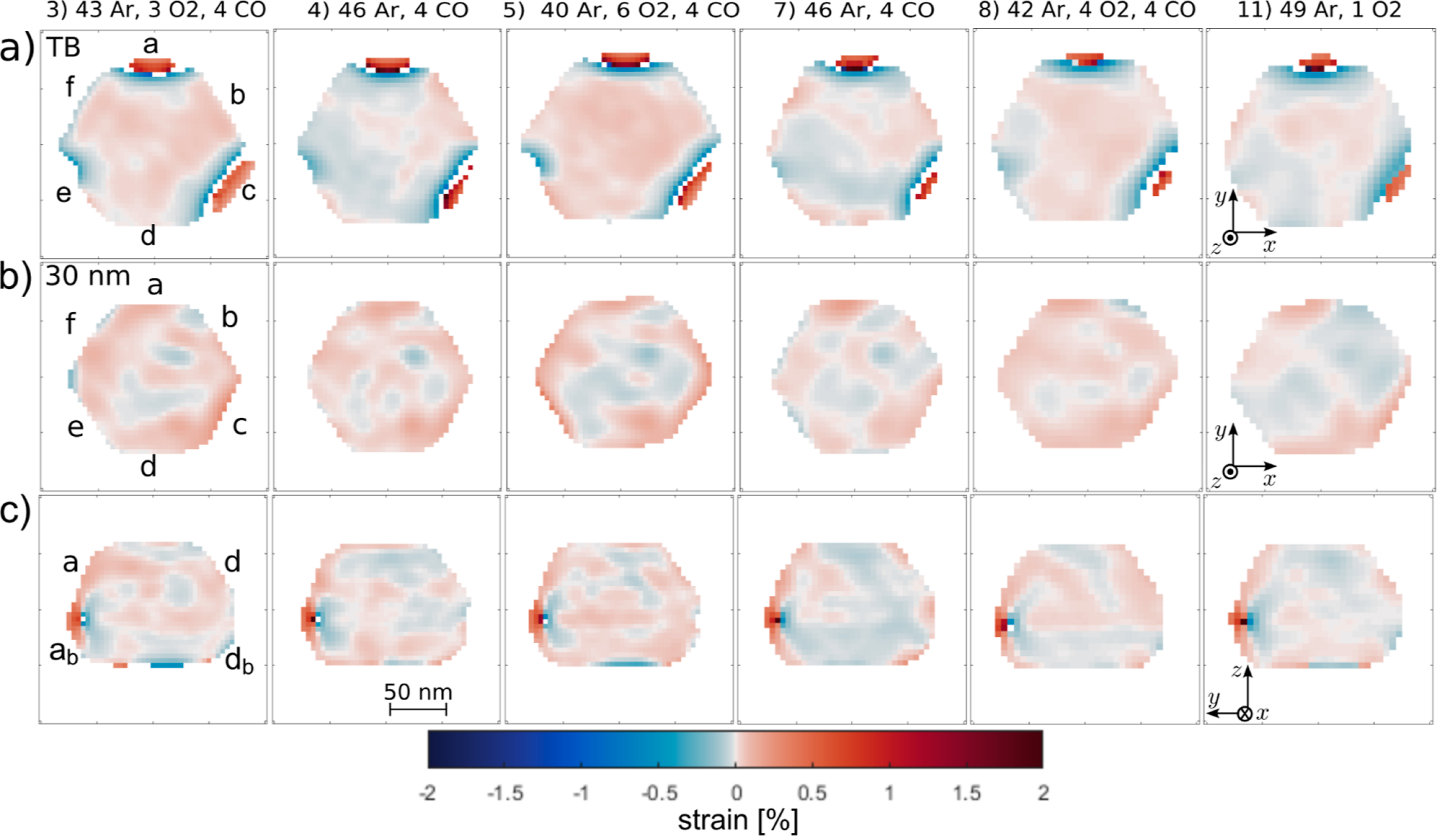
Cuts through the reconstructed strain component ϵ_zz_. Horizontal cuts with isosurface value 0.2 through the strain (a) at the twin boundary and (b) 30 nm above the twin boundary as indicated in Section S12. (c) Vertical cuts “ad”, as defined in Figure 2e. The numbers (3–11) indicate the data set number as given in Section S6 and are followed by the gas inlet in mL/min.

As one can see in Figure 6a, the dislocation lines ***t*** are parallel to the facets a $\left(t_{1}∥\left[2\overset{®}{2}0\right]\right)$ , c $\left(t_{2}∥[\overset{®}{2}02]\right)$ , and e $\left(t_{3}∥\left[02\overset{®}{2}\right]\right)$ and perpendicular to the 111 reflection. A dislocation only contributes to a diffraction contrast if the scalar product ***g*** · ***b*** ≠ 0, with Burgers vector ***b*** and reciprocal lattice vector ***g*** = (111) for the (111) Bragg peak. Thus, the observed dislocations cannot be pure screw dislocations, since the Burgers vectors for pure screw dislocations are parallel to the dislocation lines. The dislocations must therefore be rather edge or mixed dislocations. In the first case, the Burgers vector is oriented perpendicular to the dislocation line, and in the latter case, in any other angle. The displacement around the core of the dislocation could give more information about the dislocation type. Figure 6c shows the cut through the reconstructed displacement perpendicular to one of the dislocation lines (perpendicular through facets a and d, as defined in Figure 2e). The figure also indicates at which position the displacement around the core of the dislocation is taken (black circle, clock-wise direction). The displacement is plotted in Figure 6d as a function of the azimuthal angle Θ, together with the calculated displacement of a pure edge dislocation,^14^ which is

1 $$u_{x_{1}}\left(\Theta \right)=\frac{b_{1}}{2\pi }\left(\Theta +\frac{\cos\Theta \sin\Theta }{2\cdot \left(1-\nu \right)}\right)$$

with the Burgers vector magnitude *b*_1_ and Poisson ratio for 50% Pt 50% Rh ν ≈ 0.35.^46^ Unfortunately, the data are not sufficient to determine if the dislocation is a pure edge dislocation nor to analyze quantitatively the edge dislocation component of the displacement, especially since the data depends on the chosen distance *r* to the dislocation core (see Section S13). To describe the dislocation, a local coordinate system is chosen, such as ***x***_1_∥ ***b*** and ***z***_1_∥ ***t***_**1**_. By comparing the sign of the calculated strain of an edge dislocation with the reconstructed strain (see Section S13), we find that ***x***_1_ = ***b*** = (111) so ***b*** = ***g***, $y_{1}=\left(\overset{®}{2}\overset{®}{2}0\right)z_{1}=t_{1}=\left(2\overset{®}{2}0\right)$ . Thus, the missing half plane of atoms is toward the outside of the particle, as illustrated in Figure 6e. In a similar way, one can deduce the directions for the other two dislocations as well and find that between each {111} side facet, a half plane of atoms is missing, as sketched in Figure 6f. The curved shape of the dislocation line indicates that the dislocation is actually a mixed dislocation, since the Burgers vector is the same at every point in a dislocation, but the dislocation line is only parallel to ***z***_1_ between point B and C where it is a pure edge dislocation.^12,13^ The same applies to the other dislocations. Since the dislocation along facet e is not completely reaching from one corner to the other (as visual in Figure 6), the ratio of the mixed dislocation part to the pure edge dislocation part is higher compared to the other dislocations. This dislocation is also shrinking throughout the course of the experiment, as visible in Section S12. So, it is likely that starting and ending at the corners stabilized the other dislocations.

**Figure 6:**
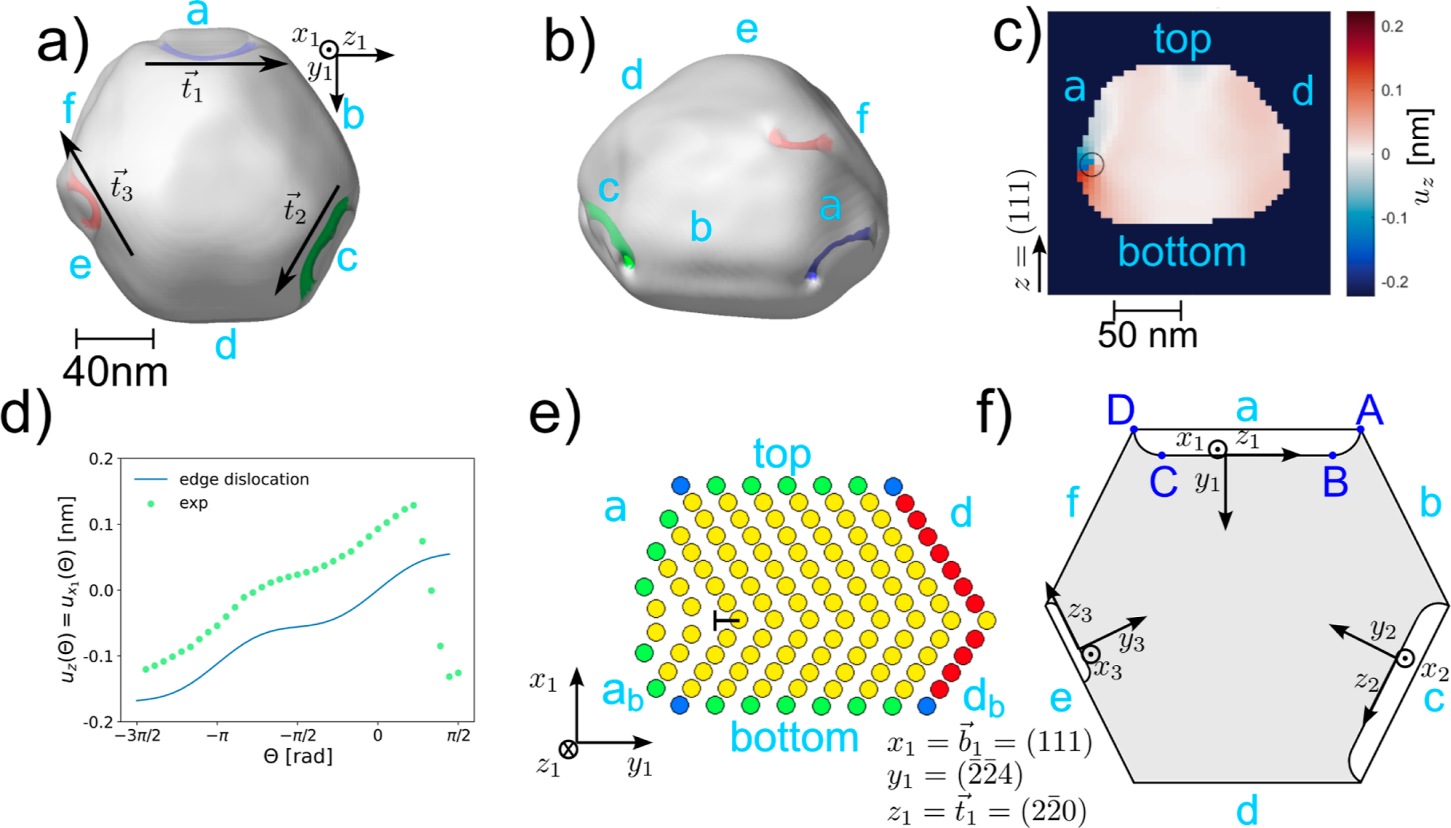
Structure of dislocations. Semitransparent 3D image of the electron density reconstructed from the first BCDI data set, measured under Ar at 430 °C. The positions of the dislocations are visible, as colored holes with an isosurface value of 0.15. (a) Top view including the dislocation line vectors ***t***_1_, ***t***_2_, ***t***_3_ and (b) side view. (c) Cut through the reconstructed displacement map, perpendicular through facets a and d. The radius of the black circle is 8.34 nm, which corresponds to two voxels. (d) Displacement as a function of the azimuthal angle Θ for the reconstructed displacement along the circle drawn in black in (c) and for the calculated displacement for an edge dislocation with ν = 0.35 and $b_{1}=0.388/\sqrt{3}$ . Note that *x*_1_ is parallel to *z*. (e) Sketch of an edge dislocation: At the border between facet a and a_b_ one part of the plane is missing (marked with T) and the atoms of the neighboring planes are shifted accordingly. (f) Sketch of the plane at the twin boundary. The missing atoms in the plane at the {111} facets a, c, and e are marked in white, so that the dislocation lines are indicated by the black lines between the white and the gray areas. The dislocation parallel to a is a pure edge dislocation between points B and C and a mixed edge-screw dislocation between points A–B and C–D. The coordinate systems are defined, so that *z*_1,2,3_ are parallel to the dislocation lines for each dislocation.

For the so described dislocations, the slip plane lies perpendicular to the twin boundary plane, since the dislocations glide in the plane defined by the dislocation lines ***t*** and the Burgers vectors ***b***. Thus, a gliding of the dislocations is prevented by the twin boundary.^47^

Comparing the strain inside the NP at the twin boundary under different gas conditions shows a higher, homogeneous strain (Ar + CO + O_2_) compared to Ar + CO or Ar + O_2_ (see Figure 5a). To quantify these changes, we defined a central region of interest (ROI) in the core of the NP, excluding the dislocations and the surface of the particle as shown in Figure 7c. At the reaction condition (data set 3), the averaged strain at the twin boundary is (0.023 ± 0.019)% high, with over 87% positive strain values (so overall tensile strain). When changing to reducing conditions (data set 4), the strain becomes more mixed (32% positive strain values) with an average compressive strain of (−0.009 ± 0.015)%. This process is reversible, as by switching back to reactive conditions (data set 5), the strain becomes more tensile again (98% positive strain values) with an average strain of (0.040 ± 0.018)%. This can be repeated over several cycles (data set 7–11, see Figure 7a). The standard deviation is a measure of the strain heterogeneity, it is much larger than the error bar of ±0.0019% from the reconstruction (see Methods section). The observed strain changes are limited to the plane at the twin boundary, as the comparison with the average strain of the central ROI of the cuts 30 nm above the twin boundary illustrate (Figure 7b, ROI defined as shown in Figure 7d). This is also confirmed by the vertical cuts through the particle (Figure 5c), since they show a line of high strain at the twin boundary under reaction conditions (data sets 3, 5, and 8). Presumably, this tensile strain is related to oxygen chemisorbed under reaction conditions.^48^ As described above, we do not observe this tensile strain under only O_2_ + Ar. Instead, we observe compression which could be related to partial oxidation of the NP.^43^ The observed compression under CO + Ar could be related to the desorption of any chemisorbed oxygen to form CO_2_, while CO does not adsorb at this temperature and pressure.

**Figure 7:**
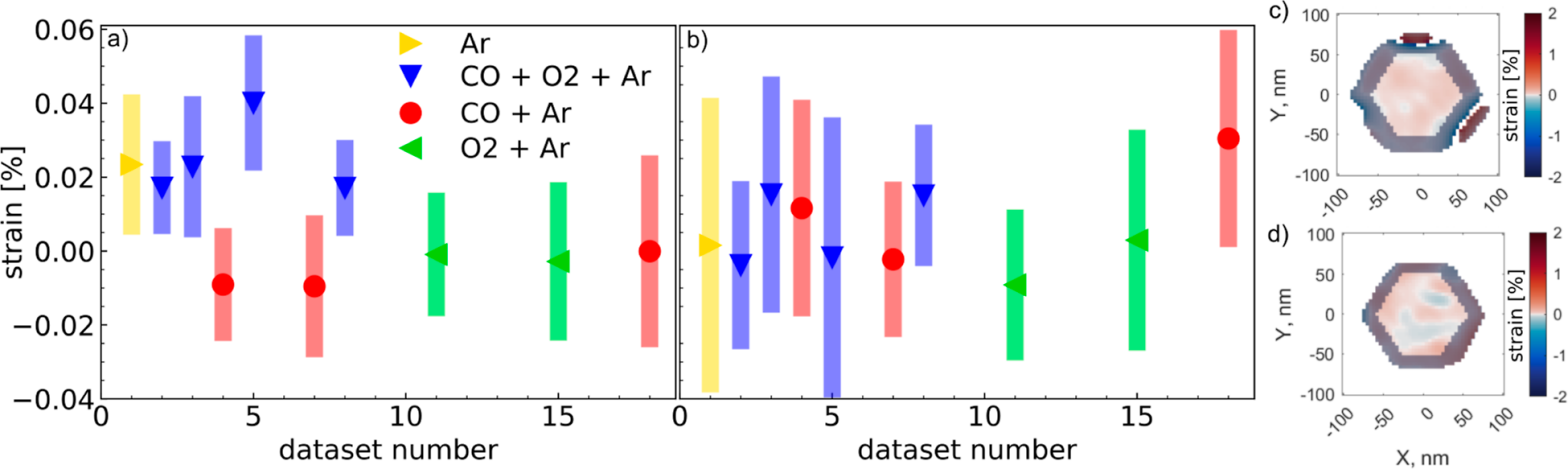
Average strain ϵ_zz_ for all data sets taken on the 111 Bragg peak (a) at the twin boundary and (b) 30 nm above the twin boundary. The bars represent the strain variation inside the ROI, indicating how homogeneously the strain is distributed, expressed as standard deviation. The average is taken over a central ROI as shown in (c) for the cut at the twin boundary and (d) for the cut 30 nm above the twin boundary, both for data set 3. All data points outside the ROI are grayed out.

## Conclusions

For improved catalyst performance, structure–activity correlations are important. The investigated PtRh nanoparticle showed a number of structural features that are expected to influence the catalytic activity. We demonstrated that BCDI is a powerful method to image the shape, strain, and defect state of catalytic nanoparticles under operando conditions. We observed dislocations along the edges of the twinned particle. They appear at the height of the Σ3 grain boundary but only at edges where the {111} facets meet. This is a unique feature induced by the reduced dimensionality and shape of the nanoparticles, since such dislocations do not appear at extended Σ3 grain boundaries in the bulk of fcc materials, which are nearly strain free. The observed dislocations can serve as adsorption sites^49^ with modified adsorption energies, especially since we observed high strain, up to 2% close to the dislocation cores. This can in turn change the adsorption energy of the adsorbed molecules and thus can influence their catalytic activity.^6,50^ As a future approach, we propose to artificially introduce such dislocations, which may allow tuning the activity of the nanoparticles. The gas atmosphere induced shape changes and formation of vicinal facets provide additional adsorption sites compared to a perfect fcc(111) particle. Overall, the preparation and investigation of particles with twin boundaries parallel to the substrate and resulting nanoparticle edge induced dislocation formation are of great interest for further studies, in which one aims to produce catalysts with controlled, active defect sites.

## Methods

### Sample Preparation

The PtRh nanoparticles were prepared by Pt and Rh codeposition on a (001) oriented STO crystal with 0.5 wt % Nb doping. The substrate was treated following a protocol including etching in a a diluted hydrofluoric acid solution (buffered oxide etch, BOE) and annealing in air.^51,52^ Furthermore, the substrate was degassed at 550 °C for 120 min in ultra high vacuum and annealed in O_2_ with a partial pressure of 4 × 10^–7^ mbar at 350 °C. The substrate cleanness was confirmed by low energy electron diffraction (LEED) and Auger electron spectroscopy (AES). Subsequently, Pt and Rh were codeposited by e-beam evaporation and molecular beam epitaxy at 850 °C, with a nominal thickness of (1.7 ± 0.4) nm and a nominal content of (58 ± 18)% Pt, see Section S1. The Pt and Rh flux values were calibrated by growing reference samples and determination of the resulting thicknesses by X-ray reflectivity; see the fitted X-ray reflectivity curves with the electron density profiles in Figure S1 in the Supporting Information. Finally, the sample was postannealed at 1100 °C for 10 min. The successful deposition of Pt and Rh was checked by LEED and AES and the composition was measured by energy-dispersive X-ray (EDX) analysis to be between 50% and 60% Pt. Additionally, the composition of the investigated NP was calculated from the *d*-spacing at 430 °C using Bragg’s and Vegard’s laws to be (50 ± 1)% Pt, see Section S5.

### Sample Characterization

SEM images were collected with a high-resolution field-emission SEM in secondary (SE) and backscatter (BSE) electron mode at an acceleration voltage of 5 kV, and the chemical composition was measured by EDX analysis.^31^ As described in previous works,^22,39^ the nanoparticle was preselected in the SEM and equipped with hierarchically arranged Pt-based markers using electron- (EBID) and ion- (IBID) beam induced deposition in the SEM and a FIB-SEM using a Pt precursor gas.

The nanoparticle height was determined by AFM before and after the BCDI experiment in tapping mode in air using an oxide-sharpened silicon cantilever with a nominal frequency of 300 kHz.^31^ The image resolution was 512 × 512 pixels, and the scanning rate was 1 Hz. A plane fit algorithm was applied to the AFM image to correct for any possible macroscopic sample tilt.

After the operando BCDI experiment, SAM from the ROI containing the nanoparticle was performed using a PHI 710 Scanning Auger Nanoprobe at the DESY NanoLab. An Oxygen (O 1s) Auger map was acquired with a pixel resolution of 256 × 256 and an energy window of 472–532 eV. The maps were collected from a 500 × 500 nm^2^ area with an electron acceleration voltage of 20 kV and a beam current of 1 nA. The data analysis routine is described in detail in Section S11 in the Supporting Information.

### Operando BCDI

The BCDI experiment was performed at the ESRF at beamline ID01 with a photon beam energy of 9 keV. The beamsize of the coherent illumination was (300 × 400) nm^2^ at the sample position with a coherent flux of 4 × 10^9^ photons/s. An incident angle between 18.63° and 19.59° was used, resulting in a footprint of around 1.1 μm. The Maxipix pixel detector with 516 × 516 pixels was mounted at a sample-to-detector distance of 0.496 m. Each pixel had a size of (55 × 55) μm^2^. To relocate the preselected nanoparticles in the X-ray beam, a fast X-ray scanning mode was used, during which the ROI of the 2D detector was read while two orthogonal piezo stages were moved to raster the sample. Choosing the ROI of the Pt 111 powder ring allowed us to relocate the nanoparticle utilizing the markers.

The 3D intensity distribution around the 111 Bragg reflection was collected by rocking the incident angle in a range of ±0.7° around the Bragg peak; see Section 4. For the asymmetric Bragg peaks (020) and (220), the in-plane angle ϕ (see Section S4) was varied in a range of ±0.5°. Typically, a step-size of 0.01° and an exposure time of 10 s per frame was used. The signal was optimized by aligning the sample position in the in-plane directions *x* and *y* every few steps of the rocking scan.

The gas environment was computer controlled by a custom-made gas dosing system as described in refs 22,39 . The applied gas mixtures are listed in Section S2. Each gas flow was set by a calibrated mass flow controller, and the total flow was 50 mL min^–1^. To correlate the BCDI measurements with the catalytic activity, a mass-spectrometer was measuring the gas composition at the outlet of the reaction chamber. The pressure of the flow reactor with a Be dome was controlled by a back pressure controller and kept constant at 0.1 bar throughout the whole experiment. The water-cooled cell was also equipped with a heater to heat the sample to 430 °C.

### Phase Retrieval

The electron density distribution of the crystalline part of the nanoparticle was reconstructed from the measured intensity distribution around the Bragg peak, using an iterative phase retrieval algorithm. Therefore, a combination of difference map (300 iterations) and error reduction (300 iterations) including shrink wrap (with the threshold of 0.2) was alternated three times for one reconstruction.^53–55^ It was averaged over ten successful reconstructions, each with a different random initial phase. The strain was evaluated in the following way: The displacement field ***u*** (***r***) is connected to the phase of the real space object by Φ­(***r***) = −***u*** (***r***)· ***g***, with the scattering vector ***g***. Thus, ***u*** (***r***) = −Φ­(***r***)/| ***h*** | = −Φ­(***r***)/(2π/*d*_*hkl*_) = −*d*_*hkl*_Φ­(***r***)/(2π) with the distance between the lattice planes in the *hkl* direction of *d*_*hkl*_ and the corresponding reciprocal lattice vector **h**. From the displacement field, one can determine the strain component ϵ_*zz*_(***r***) = ∂*u*_*z*_(***r***)/∂*z*. We define ***z*** along the (111) direction, so the scattering vector ***g*** is parallel to ***z*** in the case of the 111 Bragg peak. The error bar for the determined strain values was obtained from the standard deviation of the averaged ten reconstructions at each point inside the NP. The average over all error bars for all strain values was ±0.00179%. Together with an estimation of the error from the lattice constant calculated from the Bragg peak, the strain margin of error is ±0.0019%.

## Supplementary Material



## Notes

Calculation of nominal composition; list of gas conditions and mass spectrometer data; SEM image of markers; sketch of setup geometry; calculation of chemical composition from Bragg peak position; calculation of particle height from line-profiles; calculation of thermal misfit; all stereographic projections; sketches of surface structure of the new facets; 3D reconstruction of the strain at the new facets; description of the SAM data analysis routine to obtain the O 1s map in Figure 1; images of cuts in strain the 3D strain field; and displacement and strain around dislocation (PDF)

## ⊥

Insitute of Applied Physics, University of Tübingen, 72076 Tübingen, Germany

## #

Pohang Accelerator Laboratory, POSTECH, 37673 Pohang, South Korea.

## ¶

Constructor University, Campus Ring 1, 28759 Bremen, Germany.
