# Supplementary material for: Coherent X‑ray Diffraction Imaging of a Twinned PtRh Catalyst Nanoparticle under Operando Conditions
Source: ACS Nano. 2025 Jun 25;19(26):23552–63. doi: 10.1021/acsnano.4c15457 (PMC12257639; doi:10.1021/acsnano.4c15457)
Supplement: Supplementary file 1 [file nn4c15457_si_001.pdf]

Supplementary Information: Coherent X-ray Diffraction Imaging of a Twinned PtRh Catalyst Nanoparticle under Operando Conditions

Lydia J. Bachmann,[†,‡] Dmitry Lapkin,[¶,§] Jan-Christian Schober,[†,‡] Daniel Silvan Dolling,[†,‡] Young Yong Kim,[¶,‖] Dameli Assalauova,[¶,⊥] Nastasia Mukharamova,[†] Jagrati Dwivedi,[†] Tobias U. Schulli,[#] Thomas F. Keller,[†,‡] Ivan A. Vartanyants,[¶] and Andreas Stierle[*,†,‡]

†*Centre for X-ray and Nano Science CXNS, Deutsches Elektronen-Synchrotron DESY, Notkestraße 85, 22607 Hamburg, Germany*

‡*Department of Physics, University of Hamburg, Notkestraße 9-11, 22607 Hamburg, Germany*

¶*Deutsches Elektronen-Synchrotron DESY, Notkestraße 85, 22607 Hamburg, Germany*

§*Present Adress: Insitute of Applied Physics, University of Tübingen, 72076 Tübingen, Germany*

‖*Present address: Pohang Accelerator Laboratory, POSTECH, 37673 Pohang, South Korea*

⊥*Present address: Constructor University, Campus Ring 1, 28759 Bremen, Germany*

#*European Synchrotron Radiation Facility (ESRF), 38043 Grenoble Cedex 9, France*

E-mail: andreas.stierle@desy.de

S1 Calculation of the nominal composition

The nominal thickness and composition are calculated from the deposition rate for each material. Therefore, two calibration samples were grown on α-Al_2O_3(0001), one for each material. The deposition time for the Pt sample was 45 min with the value of the flux monitor of 22 nA. The X-ray reflectivity (XRR) curve shown in Figure S1a) was fitted with the Python package `xraytutilities`,[1] using a two layer fit with a particle height d_1 of 12.9 Å with a coverage Φ_1 of 16% and d_2 of 9.96 Å with a coverage Φ_2. Thereby, the coverages $\Phi_{1,2}$ are calculated by the real part of the fitted index of refraction $\delta_{\text{fit}1,2} \cdot 2$ divided by the real part of refraction index of the bulk δ_{bulk}.[2] With this, the nominal thickness d_{nominal}, equivalent to the height of the same amount of material distributed as a layer on the substrate, can be calculated by

$$d_{\text{nominal}} = d_1 \cdot \Phi_1 + d_2 \cdot \Phi_2 = 12.898\,\text{Å} \cdot 0.16 + 9.96\,\text{Å} \cdot 0.02 = 2.26\,\text{Å} \tag{1}$$

with the error of

$$\Delta d_{\text{nominal}} = \Phi_1 \cdot \Delta d_1 + \frac{2 \cdot d_1}{\delta_{\text{bulk}}} \Delta\delta_{\text{fit1}} = 0.04\,\text{Å} \tag{2}$$

With the evaporation time t and the value of the flux monitor f, the deposition rate s was calculated with:

$$s = \frac{d_{\text{nominal}}}{t \cdot f} = 2.08 \times 10^{-3} \frac{\text{Å}}{\text{min nA}} \tag{3}$$

with the error

$$\Delta s = \frac{1}{t \cdot f} \Delta d_{\text{nominal}} + \frac{d_{\text{nominal}}}{t \cdot f^2} \Delta f = 0.13 \times 10^{-3} \frac{\text{Å}}{\text{min nA}} \tag{4}$$

In analogy, the deposition rate for Rh is calculated from a sample grown for 50 min with a value of the flux monitor of 20 nA. As shown in Figure S1b), a model with two layers of Rh is used to fit the XRR curve of this calibration sample, which leads to a deposition rate of

(0.0065 ± 0.0011)Å$/(\min \times \text{nA})$.

Knowing the deposition rate for each element, the nominal thickness of each element can be calculated to:

$$d_{\text{Pt}} = s_{\text{Pt}} \cdot t \cdot f_{\text{Pt}} \quad = \quad 9.9\,\text{Å} \tag{5}$$

$$d_{\text{Rh}} = s_{\text{Rh}} \cdot t \cdot f_{\text{Rh}} \quad = \quad 7.1\,\text{Å} \tag{6}$$

with the errors

$$\Delta d_{\text{Pt}} = t \cdot f_{\text{Pt}} \cdot \Delta s_{\text{Pt}} + t \cdot s_{\text{Pt}} \cdot \Delta f_{\text{Pt}} \quad = 1.0\,\text{Å} \tag{7}$$

$$\Delta d_{\text{Rh}} = t \cdot f_{\text{Rh}} \cdot \Delta s_{\text{Rh}} + t \cdot s_{\text{Rh}} \cdot \Delta f_{\text{Rh}} \quad = 2.6\,\text{Å} \tag{8}$$

So the total nominal thickness is $d_{\text{tot}} = d_{\text{Pt}} + d_{\text{Rh}} = 17\,\text{Å} = 1.7\,\text{nm}$ with $\Delta d_{\text{tot}} = \Delta d_{\text{Pt}} + \Delta d_{\text{Rh}} = 3.6\,\text{Å}$ and the nominal Pt composition is $d_{\text{Pt}}/d_{\text{tot}} = 0.58$ with the error $\Delta(d_{\text{Pt}}/d_{\text{tot}}) = d_{\text{Pt}}/d_{\text{tot}} + d_{\text{Pt}}/d_{\text{tot}} \cdot \Delta d_{\text{tot}} = 0.18$.

The XRR measurements were performed in a 6-circle diffractometer[3] at the DESY NanoLab laboratory with a $\text{CuK}_{\alpha_{1,2}}$ source so a wavelength of 154 pm, a beam size of 1 mm x 1 mm and a distance between sample and source of 0.48 m.

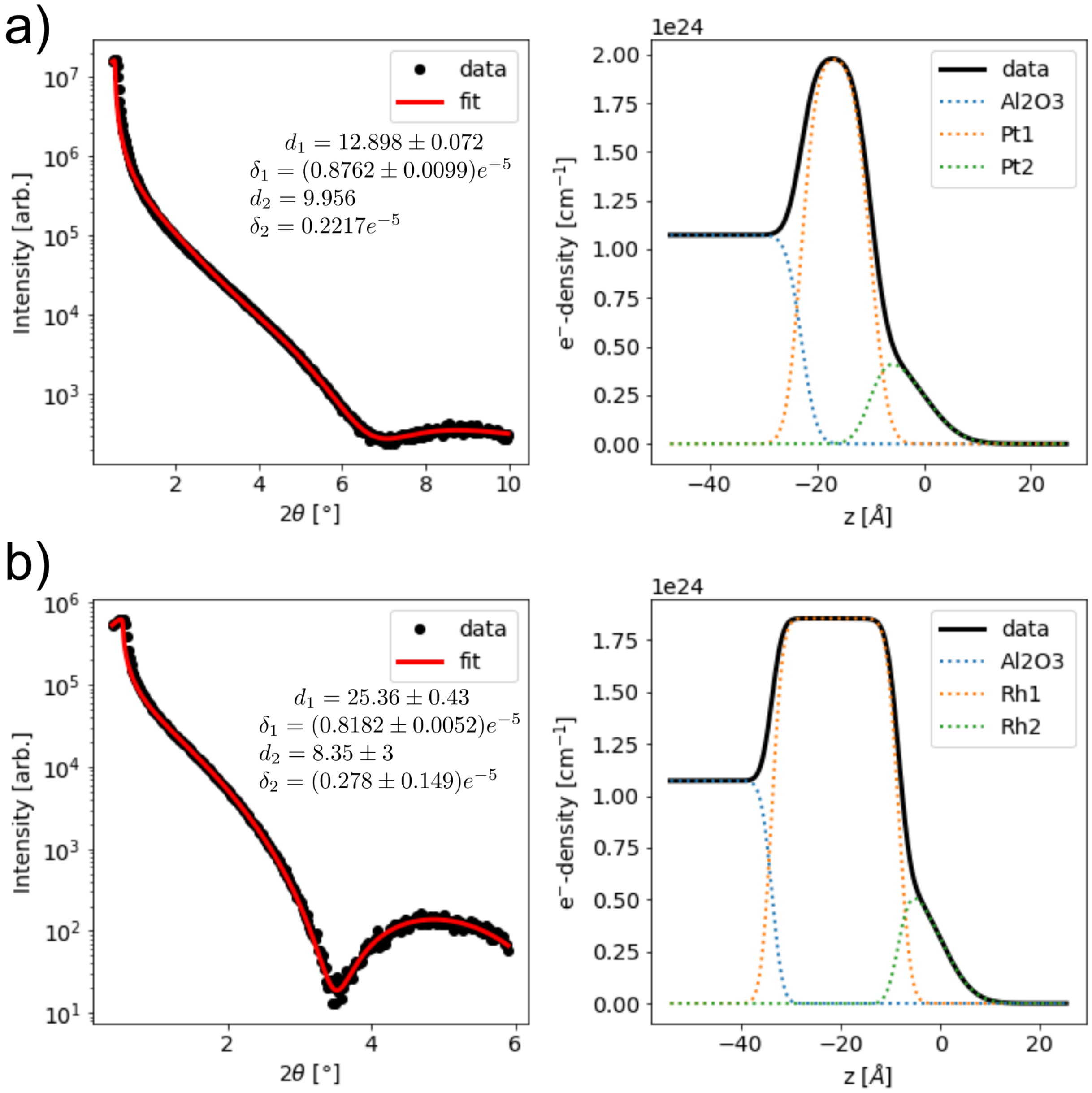

Figure S 1: **XRR curves for the calibration samples (left) and electron density profile obtained from the fit (right).**
a) XRR curve with fit for Pt. **b)** XRR curve with fit for Rh.

S2 Gas conditions

Figure S2 shows the mass spectrometer data. During the CO oxidation, the CO and O_2 signal are reduced due to mass transfer limitations of the set-up, indicating the high activity of the sample. Table 1 shows all gas conditions applied to the sample and the number of the BCDI dataset, if a reconstructable dataset was acquired.

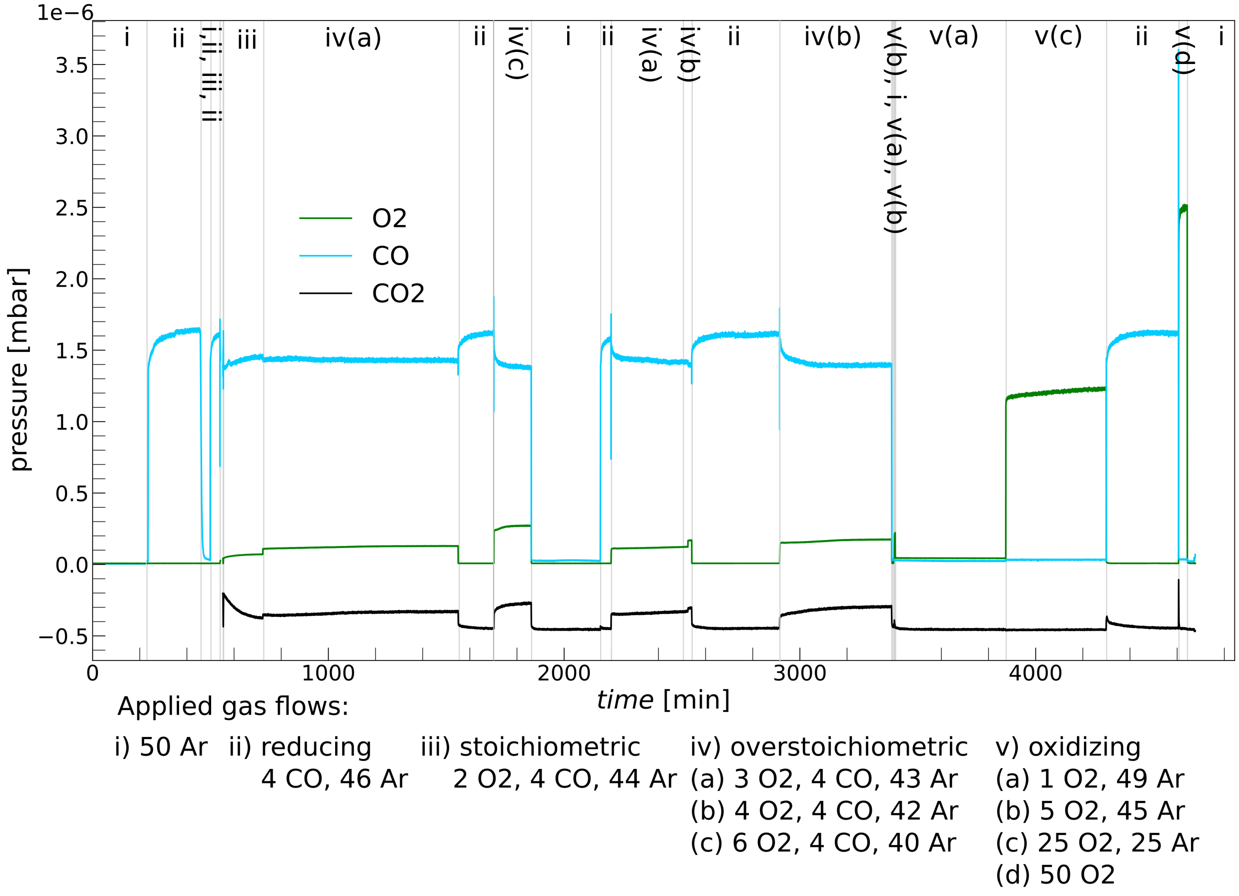

Figure S 2: **Masspectrometer data.** The CO_2 signal is amplified by a factor of ten and shifted by -0.5×10^{-6} mbar for clarity. The gas flows are given in [mL min^{-1}].

Table S 1: **Gas conditions before, during and after the *operando* BCDI experiment.**

	temperature [°C]	Ar [mL min^{-1}]	O_2 [mL min^{-1}]	CO [mL min^{-1}]	H_2 [mL min^{-1}]	dataset
Cleaning	350	25	-	-	25	-
i) pure Ar	430	50	-	-	-	1)
ii) reducing	430	46	-	4	-	-
i) pure Ar	430	50	-	-	-	-
ii) reducing	430	46	-	4	-	-
iii) stoichiometric	430	44	2	4	-	-
ii) reducing	430	46	-	4	-	-
iii) stoichiometric	430	44	2	4	-	2)
iv)a overstoichiometric	430	43	3	4	-	3)
ii) reducing	430	46	-	4	-	4)
iv)c overstoichiometric	430	40	6	4	-	5)
i) pure Ar	430	50	-	-	-	-
ii) reducing	430	46	-	4	-	-
iv)a overstoichiometric	430	43	3	4	-	-
iv)b overstoichiometric	430	42	4	4	-	-
ii) reducing	430	46	-	4	-	6),7)
iv)b overstoichiometric	430	42	4	4	-	8)-10)
v)b oxidizing	430	45	5	-	-	-
i) pure Ar	430	50	-	-	-	
v)a oxidizing	430	49	1	-	-	-
v)b oxidizing	430	45	5	-	-	-
v)a oxidizing	430	49	1	-	-	11),13)
v)c oxidizing	430	25	25	-	-	14)-17)
ii) reducing	430	46	-	4	-	18)-20)
v)d oxidizing	430	-	50	-	-	-
i) pure Ar	430	50	-	-	-	-

S3 Hierarchical marker arrangement for nanoparticle relocation

To be able to track the nanoparticle before, during and after the catalysis experiment, the particle was marked with Pt based markers as shown in Figure S3 by ion beam induced deposition (IBID), using a dual beam FIB/SEM instrument.

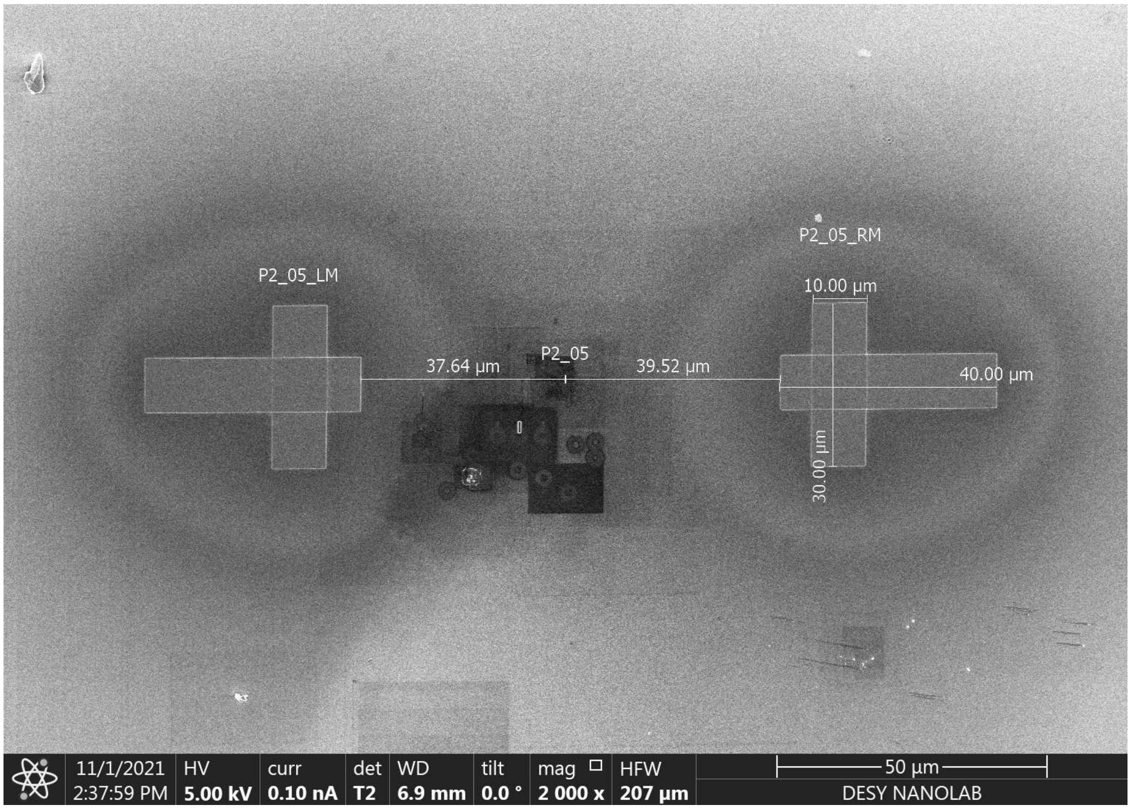

Figure S 3: **Marker to track the nanoparticle.**
To track the particle 'P2_05' during the catalysis experiment and to be able to analyze the same particle before and after the catalysis experiment by SEM, AFM and SAM in a correlative approach, the particle was marked by markers (labeled 'P2_05_LM' and 'P2_05_RM'). The relative distances from the nanoparticle to the markers are given in the image as well as the size of the markers.

S4 Geometry of the BCDI experiment

The geometry of the set-up in real space including exemplary detector images for all three reflections is shown in Figure S4.

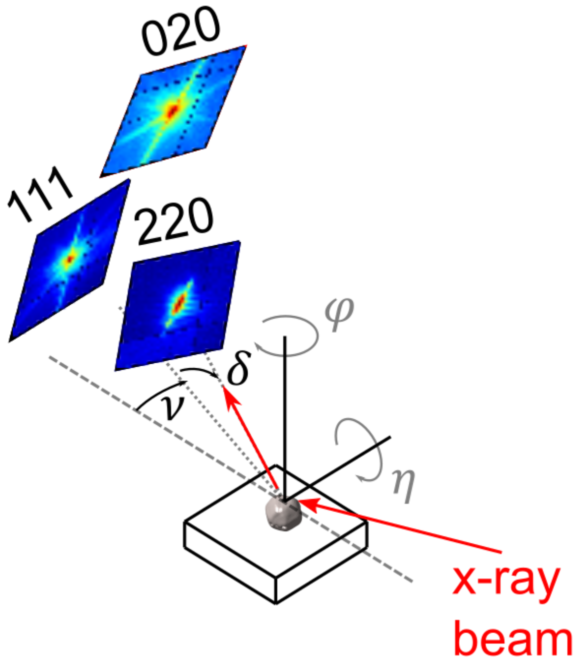

Figure S 4: **Sketch of the experimental geometry.** The X-ray beam is focused by Fresnel zone plates (FZP) onto a single nanoparticle. The Bragg coherent diffraction pattern is collected by a 2D detector, which is positioned at an angle δ (out-of-plane angle) and ν (in-plane angle) depending on the measured reflection 111, 020 or 220. To collect one BCDI dataset, the intensity distribution around the Bragg peak was scanned by rocking the sample tilt in out-of-plane direction (η) for 111 and in in-plane direction (φ) for 020 and 220. During the angular scan, the sample was tracked at every step of the rocking curve.

S5 Chemical composition calculated from the Bragg peak position

The average chemical composition from the investigated nanoparticle can be calculated from Vegard's law

$$a_{\text{alloy}} = x \cdot a_{\text{Pt}} + (1 - x)a_{\text{Rh}} \tag{9}$$

with a_{Pt} =3.94 Å and a_{Rh} =3.82 Å at 430 °C.[4,5] The mean lattice spacing a_{alloy} is calculated by

$$a_{\text{alloy}} = \frac{\sqrt{h^2 + k^2 + l^2} \cdot \lambda}{2 \cdot \sin(\Theta)} \tag{10}$$

with the wavelength $\lambda = 1.378$ Å and the 111 reflection (thus $h = k = l = 1$). The angle between the incident and the scattered beam 2Θ was calculated from the out-of-plane detector angle δ and the in-plane detector angle ν, taking the sample-detector distance SDD and the in- and out-of-plane distance between the Bragg peak and the direct beam position $d_{\text{in-plane}}$ and $d_{\text{out-of-plane}}$ into account:

$$2\Theta = \arccos(\cos(\delta + \arctan(\frac{d_{\text{out-of-plane}}}{SDD})) \cdot \cos(\nu - \arctan(\frac{d_{\text{in-plane}}}{SDD}))) \tag{11}$$

Averaging all Bragg peak positions from the 10 collected (111) reflection datasets leads to a lattice constant of 3.879 Å±0.001 Å, thus a concentration of $(50 \pm 1)\%$ Rh at 430°C. This agrees well with the concentration gradient of $40\% - 50\%$ Rh measured by energy dispersive X-ray analysis (EDX) on a simultaneously prepared sample, corresponding to 50-60% Pt reported in the main part of the manuscript.

S6 Height calculated from line-profiles of BCDI datasets

The size of the nanoparticle was determined from the 3D diffraction pattern in reciprocal space. Therefore, all detector images were flatfield corrected, stacked and hot pixels were set to -1. Then the resulting 3D diffraction pattern was transformed into the laboratory coordinate system. An example of such a 3D diffraction pattern is shown in Figure S5a). From a line-profile along the fringes in Qz-direction one can calculate the distances dQ between the minima (green dots in Figure S5a) inlet), which allows determining the height h of the nanoparticle by the following equation:

$$h = \frac{2\pi}{\overline{dQ}} = \frac{2\pi}{\frac{1}{n}\sum dQ} \tag{12}$$

So it is averaged over all n distances dQ with the standard deviation $s_{\overline{\mathrm{dQ}}}$, which leads to an error for the height of:

$$s_{\mathrm{h}} = \frac{2\pi}{\overline{dQ}^2} s_{\overline{\mathrm{dQ}}} \tag{13}$$

To reduce statistical errors and to avoid gaps in the profile due to removed pixels, each line-profile is averaged over 5 voxels and normalized, while taking into account that the fringes are not exactly parallel to Qz. All line-profiles are shown in Figure S5b). As shown in table S2, the heights of the top part (020 reflection) plus the height of the bottom part (220 reflection) agrees well with the height of the total particle (111 reflection). Throughout the experiment, no height change was detected within the error of measurement.

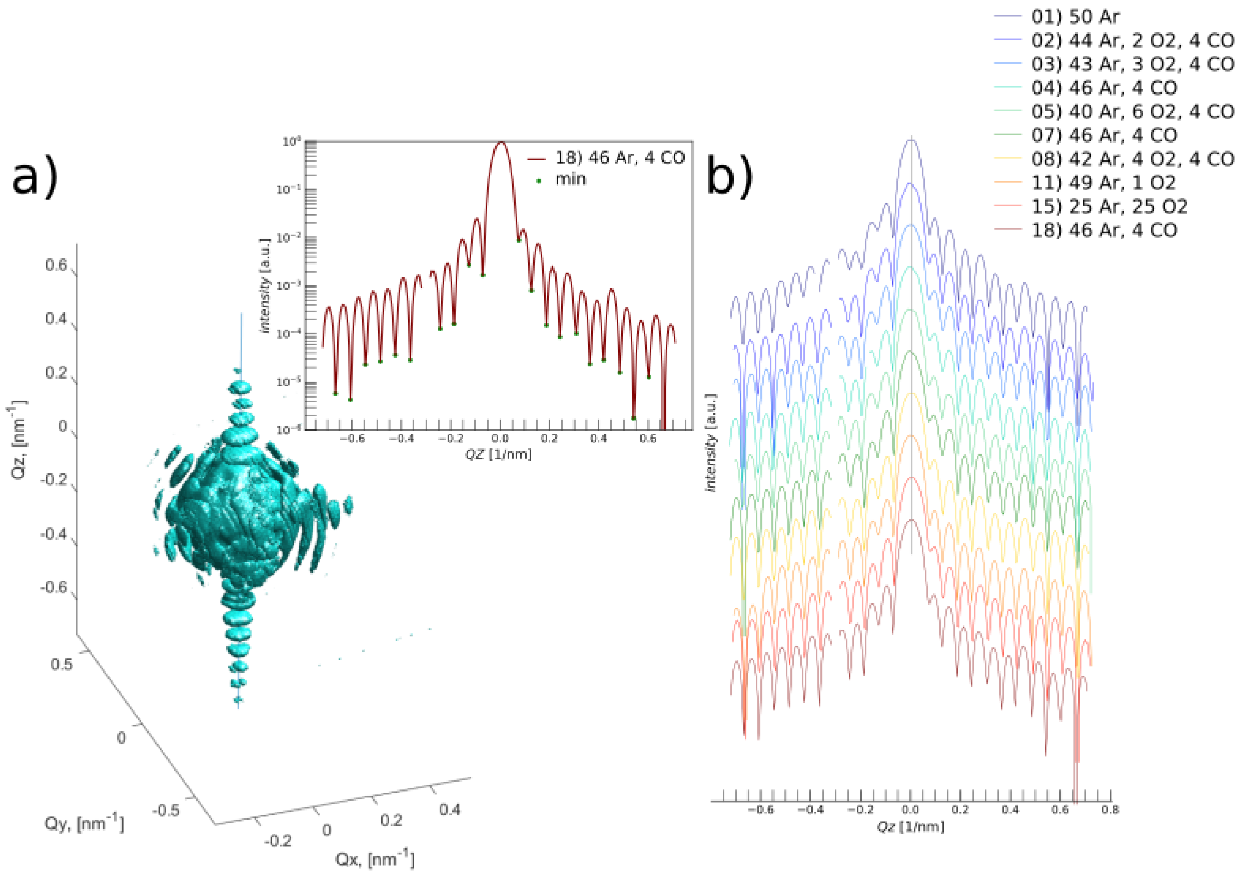

Figure S 5: **3D diffraction pattern of BCDI dataset 18 with line-profile and overview over all line-profiles.**

a) 3D diffraction pattern of the last (111) dataset 18, at 46 mL min^{-1} Ar and 4 mL min^{-1} CO. The inlet shows the line-profile averaged over the five blue lines in a). These lines have the same tilt but are shifted by one voxel. The green dots mark the local minima. **b)** Plot of all line-profiles through (111) patterns, similar as shown in a). They are normalized individually and shifted in x to be centered at 0 (vertical gray line). For better visibility, they are shifted in y and a semi-logarithmic scale was chosen. The gap on the left side is due to the detector gap.

Table S 2: **Nanoparticle height calculated from the distances between the fringes in Qz-direction.**
All heights were calculated over an average of five line-profiles, while their tilt was adapted for each dataset as shown in Figure S5. The error is the standard deviation s_{h} from averaging over all distances dQ and the error for the height from the 220 Bragg peak plus the height from the 020 Bragg peak is $s_{\mathrm{h(020)+h(220)}} = \sqrt{s^2_{\mathrm{h(020)}} + s^2_{\mathrm{h(220)}}}$. The standard deviations of the heights of the $(220)_{\mathrm{bottom}}$ Bragg peak are smaller than for the $(020)_{\mathrm{top}}$ and (111) peaks and could sometimes not be calculated, because only 2-4 neighboring minima could be recorded. The entries are sorted according to the order in which they were taken, with the first dataset at the top. The heights listed in the same line are taken without changing the gas conditions in between.

dataset	gas condition [$\mathrm{mL\,min^{-1}}$]	$\mathrm{h(020)_{top}}$ [nm]	$\mathrm{h(220)_{bottom}}$ [nm]	$\mathrm{h(020)_{top}}$ + $\mathrm{h(220)_{bottom}}$ [nm]	height (111) [nm]
1)	50 Ar	-	-	-	105 ± 7
2)	44 Ar, 2 O2, 4 CO	-	-	-	106 ± 8
3)	43 Ar, 3 O2, 4 CO	-	-	-	106 ± 8
4)	46 Ar, 4 CO	-	-	-	106 ± 8
5)	40 Ar, 6 O2, 4 CO	-	-	-	105 ± 7
6)-7)	46 Ar, 4 CO	66 ± 2	-	-	105 ± 9
8-10)	42 Ar, 4 O2, 4 CO	66 ± 4	41 ± 1	107 ± 4	105 ± 5
11)-13)	49 Ar, 1 O2	67 ± 2	40	106	107 ± 6
14)-17)	25 Ar, 25 O2	67 ± 3	41 ± 1	108 ± 3	106 ± 7
18)-20)	46 Ar, 4 CO	67 ± 3	40	107	106 ± 7

S7 Calculation of thermal misfit

The lattice constants of a PtRh nanoparticle with (50 ± 1)% Rh and the misfit between that nanoparticle and the STO support at room temperature (RT), the temperature of the *operando* experiment (430 °C), the overgrowth temperature (850 °C) and the temperature at which the sample got post-annealed (1100 °C) are listed in table S3. The increase of the misfit when cooling down from 1100 °C to RT may have induced a strain high enough to induce the stacking inversion.

Table S 3: Lattice constants at RT, 430 °C, 850 °C and 1100 °C for Pt (a_{Pt}), Rh (a_{Rh}), (50 ± 1)% Rh with (50 ± 1)% Pt (a_{PtRh}) and STO (a_{STO}).[4–6] Additionally, the misfit between a_{PtRh} and a_{STO} was calculated.

T [°C]	a_{Pt} [nm]	a_{Rh} [nm]	a_{PtRh} [nm]	a_{STO} [nm]	misfit [%]
RT	3.924	3.803	3.864±0.001	3.905	1.07±0.03
430	3.939	3.819	3.879±0.001	3.920	1.06±0.03
850	3.957	3.836	3.897±0.001	3.935	0.98±0.03
1100	3.969	3.849	3.909±0.001	3.943	0.89±0.03

S8 Stereographic projections

To identify the first appearance of the new facets, stereographic projections were made from the reconstructed 3D images of the (111) datasets. Stereographic projections are perspective projections of a sphere onto a plane, so that the central spot corresponds to the top (111) surface. As visible in Figure S6 the first new facet appears for the first time in the projection of dataset 7 under CO + Ar. The gas environments between the previous dataset 5 (overstoichiometric condition) and dataset 7 (reducing condition) are listed in table S1 (dataset 6 was taken at the same condition as 7, but on the asymmetric Bragg peak). Numerous orientations for this new facet were tested by calculating the average distance between the spot of the new facet and the facet orientation by $\sqrt{\alpha^2_{\text{azimuth}} + \alpha^2_{\text{radial}}}$. This distance would be zero for a perfect match between the measured facet and the assumed facet orientation. For all calculations dataset 15 is neglected, because there the new spot is split. The error bar is the standard deviation from the average.

Possible orientations were taken from literature[7,8] and higher index facets were considered as well. Calculating the averaged angular distance shows that the facet orientation $42\bar{1}$ describes best the new facet I with $\sqrt{\alpha^2_{\text{azimuth}} + \alpha^2_{\text{radial}}} = 3.6° \pm 1.3°$, followed by $32\bar{1}$ ($6.7° \pm 2.8°$) and $52\bar{1}$ ($8.29° \pm 2.7°$), see Figure S6 dataset 7. The more commonly reported facet $31\bar{1}$[7,9] has a higher averaged angular distance with $11.4° \pm 2.6°$. In principle one is assuming that lower indexed facets are preferable, but nevertheless higher index facts have been reported as well.[10]

The second new facet II appears at dataset 15 (oxidizing conditions), directly after dataset 11 (oxidizing conditions). For the second new facet none of the facet orientations tested for the first new facet fits. Instead a facet orientation of $7\bar{1}2$ fits perfectly ($0.6° \pm 0.2°$), the lowest index facet close to the observed facet was the $4\bar{1}1$ orientation ($5.9° \pm 0.5°$).

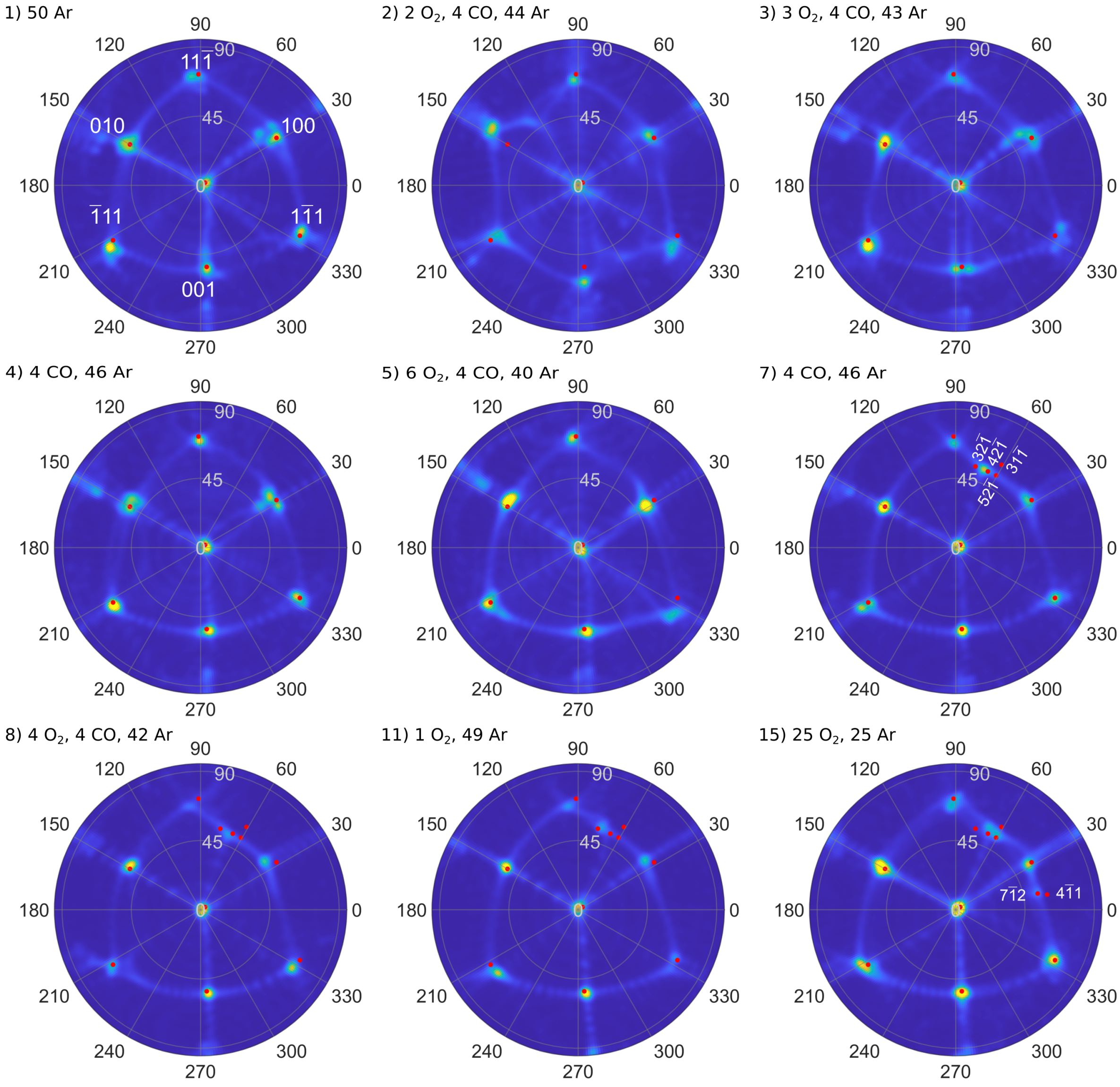

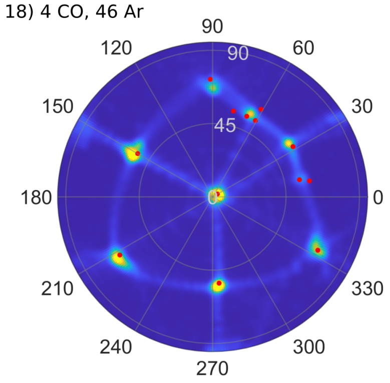

Figure S 9: Stereographic projection for all (111) datasets. The numbers refer to the number of the dataset, while the gasflow is given in [mL/min]. The azimuth corresponds to the azimuth around the nanoparticle and the radius is the angle between the top facet and the sidefacets. Red dots are marking the expected positions for each facet, the orientation of the facets are given in white at their first appearance. The new facet I appears first for dataset 7 and the second new facet II appears first for dataset 15.

S9 Surface structure of the new facets

This section presents possible surface structures for the new facets, as described in section S10. Additionally, the initial facets of the nanoparticle are shown as well.

In Figure S7 surface models of selected surface orientations of the first new facet are shown. Figure S7a) shows the $42\bar{1}$ facet, Figure S7b) shows the $32\bar{1}$ facet and Figure S7c) shows the $52\bar{1}$ facet. So all three facets that are found to be in good agreement with the new facet I have {111} oriented terraces with monoatomic {111} + {100} faceted steps. They only differ in the number of atoms in the {100} part of the steps (2 atoms for $32\bar{1}$, 3 atoms for $42\bar{1}$ and 4 atoms for $52\bar{1}$). In contrast the more commonly found facet $31\bar{1}$ has straight steps, consisting of monoatomic {111} faceted step and monoatomic {100} faceted step as shown in Figure S7d).

In Figure S9, selected surface orientations of the second new facet are shown. Figure S9a) shows the $42\bar{1}$ surface, which consists of one 2 atoms and one 3 atoms wide {100} terraces with monoatomic {111}-faceted steps. Figure S9b) shows the $7\bar{1}2$ surface, which consists of one 2-3 atoms wide {100} terrace with a monoatomic 'zig-zag' shaped step ({101} + {111} faceted). So both possible facets consist of {100} oriented terraces and {111} faceted steps, although the step edge of the $7\bar{1}2$ also includes {101} faceted steps.

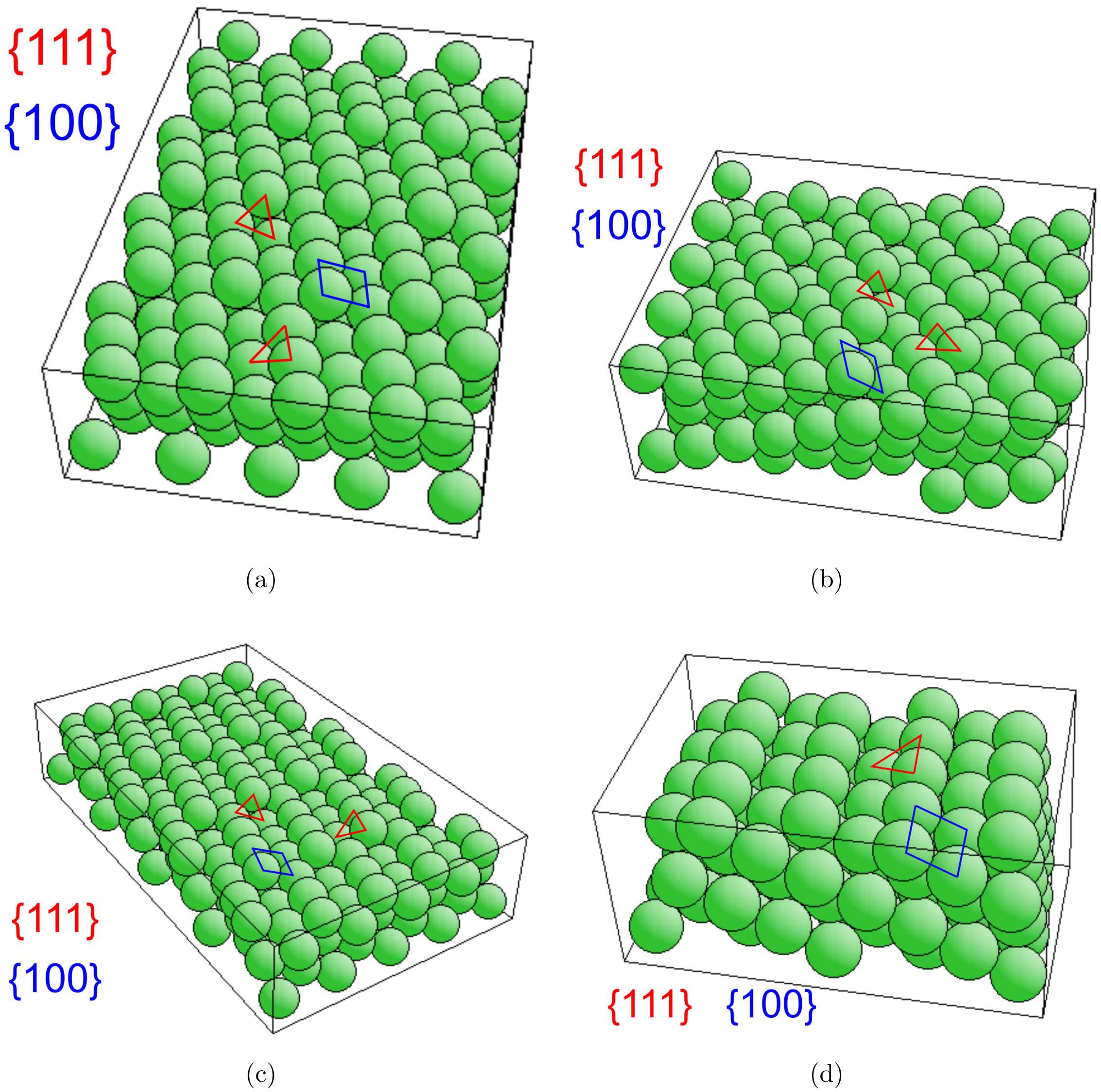

Figure S 7: Models of possible surface orientations of the first new facet: a) $42\bar{1}$ b) $32\bar{1}$ c) $52\bar{1}$ d) $31\bar{1}$. The models are made with B.A.L.S.A.C.[11]

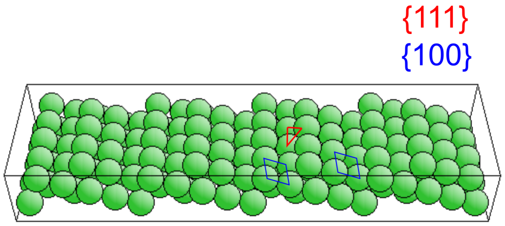

(a)

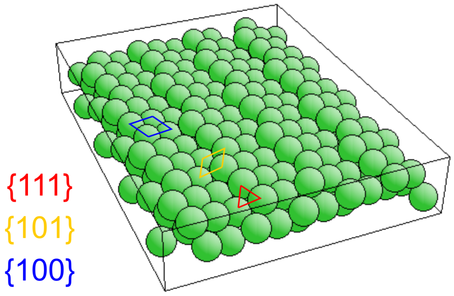

(b)

Figure S 8: **Models of possible surface orientations of the second new facet:** a) $4\bar{1}1$ b) $7\bar{1}2$. The models are made with B.A.L.S.A.C.[11]

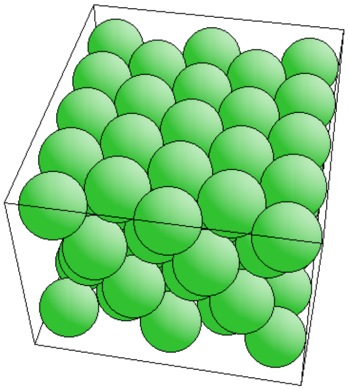

(a)

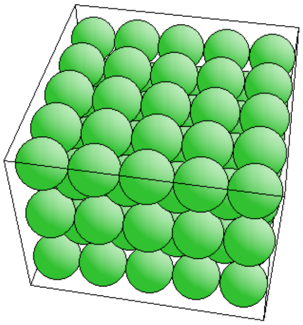

(b)

Figure S 9: **Models of the facets with the orientations:** a) 111 b) 100. The models are made with B.A.L.S.A.C.[11]

S10 Strain at new facets

Figure S10 shows an overview over the development of the strain with view on the new facet.

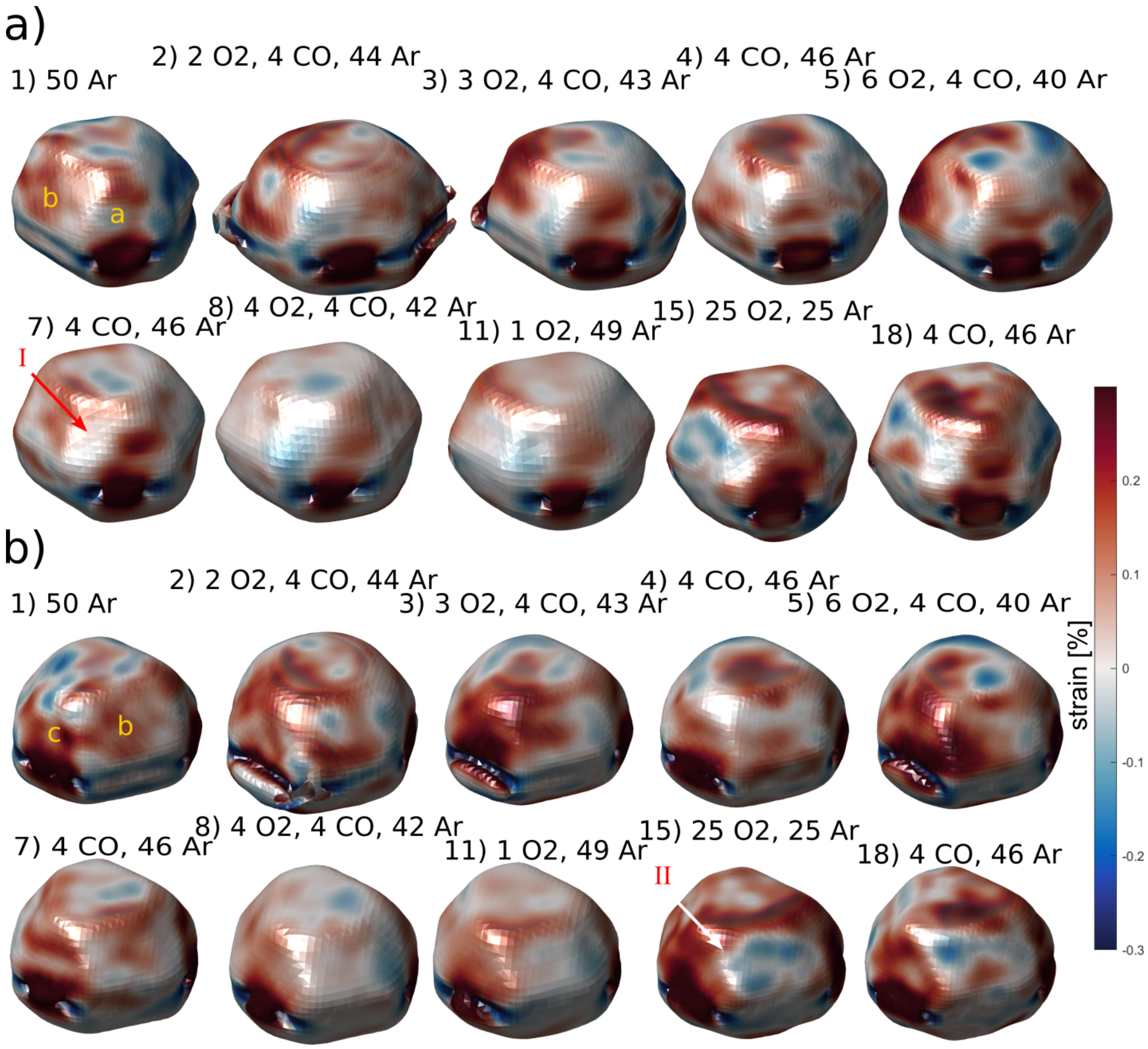

Figure S 10: **Strain evolution during facet formation.**
a) View on the new facet I. b) View on the new facet II.

S11 Scanning Auger Microscopy

The Auger maps were acquired using a PHI 710 Scanning Auger Nanoprobe with a pixel resolution of 256×256 and an energy window of $472 - 532$ eV for Oxygen, $266 - 326$ eV for Rhodium and $1927 - 1987$ eV for Platinum.[3] The maps were collected from a 500×500 nm^2 area with an electron acceleration voltage of 20 kV and a beam current of 1 nA. For quantitative analysis, the Auger maps were processed and analyzed using CasaXPS.[12] Spectra corresponding to each individual pixel were extracted. However, these raw spectra exhibit high signal-to-noise ratio, making direct analysis challenging. To overcome this, the spectra were processed using the linear least squares (LLS) fitting method. LLS fitting utilizes a principal component analysis by decomposing the experimental spectrum into a linear combination of the most dominant spectra, minimizing residual errors. Following the LLS fitting, background subtraction was performed on each pixel spectrum to quantify the oxygen signals. The quantified regions were then compiled and converted into a spatial image, representing the elemental distribution across the scanned area. The combination of LLS fitting and background subtraction enhanced the accuracy of the map, facilitating clearer interpretation of the elemental distribution. A combination of Gaussian and Lorentzian functions was employed for peak fitting to accurately represent the spectral features.

The analysis of the O map revealed that the main intensity was concentrated at the center of the particle, while lower intensities were detected at the edges and substrate, indicating a non-uniform distribution of the O element across the sample. The scanning Auger O map in Figure 1h) furthermore indicates a high oxygen level on the agglomerate in the SAM-SEM image in Fig 1g) indicated by the white arrow. The evaluation of the Pt map in Figure S11a) showed a slightly lower intensity in the center of the particle and the analysis of the Rh map in Figure S11b) revealed that the intensity was lower in the center of the particle, as well as at the agglomerate.

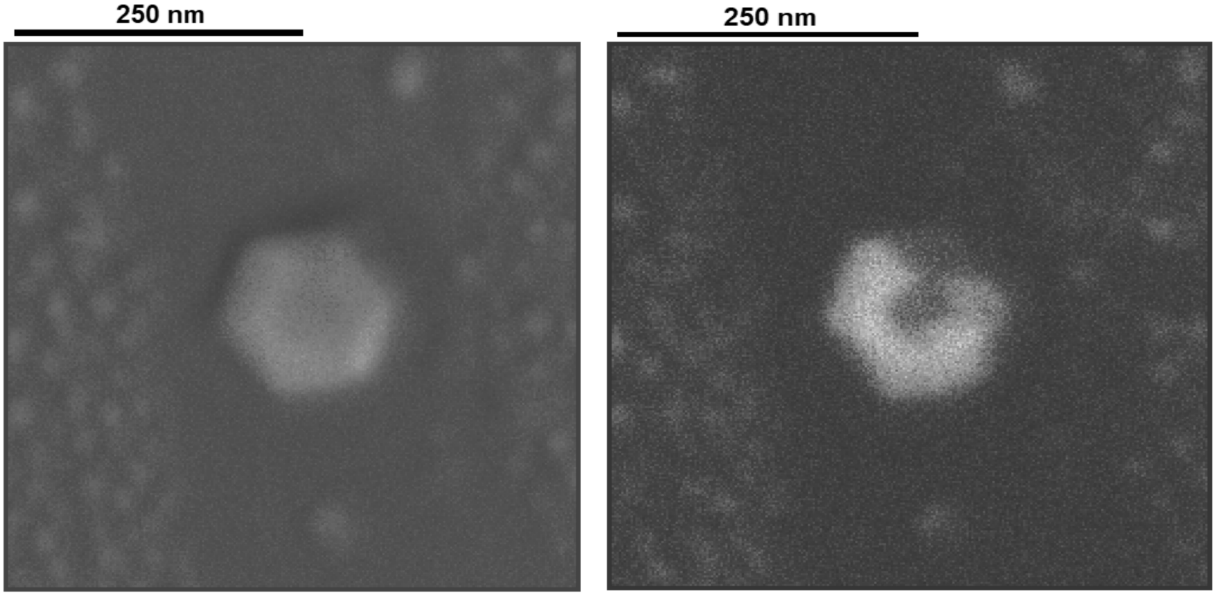

Figure S 11: **Scanning Auger maps of Pt and Rh.**
SAM map of a) Pt and b) Rh, the data was treated as described in the text.

S12 Strain cuts

The cuts in Figure S12 show the strain component ϵ_{zz} in-plane with the twin boundary and 30 nm above the twin boundary for all datasets taken. The increase of the strain under reaction conditions (dataset 2, 3, 5 and 8) at the twin boundary is visible.

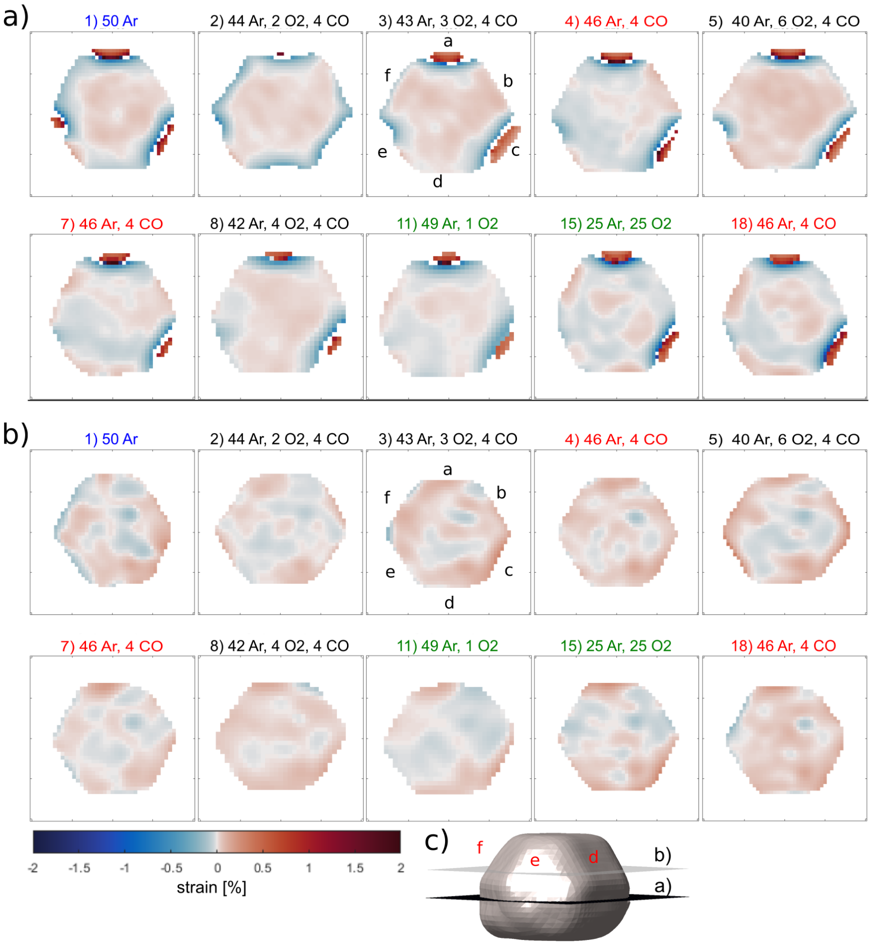

Figure S 12: **Horizontal cuts** a) at the twin boundary and b) 30 nm above the twin boundary as indicated in c).

S13 Edge dislocation

Figure S13a) shows the reconstructed displacement $u_z(\Theta)$ around the dislocation parallel to facet a of dataset 1 with different distances r to the dislocation core. The displacement of an edge dislocation can be calculated by

$$u_{x_1}(x_1, y_1) = \frac{b_1}{2\pi}(\arctan\frac{y_1}{x_1} + \frac{x_1 y_1}{2 \cdot (1-\nu)(x_1^2 + y_1^2)}) \tag{14}$$

Figure S13b) shows a cut through the reconstructed strain ϵ_{zz} and c) the calculated strain of an edge dislocation with

$$\epsilon_{x_1x_1}(x_1, y_1) = \frac{\partial u_{x_1}}{\partial x_1} = \frac{b_1}{2\pi}(\frac{-y_1}{x_1^2 + y_1^2} + \frac{y_1(y_1^2 - x_1^2)}{2(1-\nu)(x_1^2 + y_1^2)^2}) \tag{15}$$

The coordinate system x_1, y_1, z_1 is defined for this edge dislocation, following the standard literature nomenclature for the description of edge dislocations, with $x_1 \parallel \boldsymbol{b_1}$ and $z_1 \parallel \boldsymbol{t_1}$. The coordinate system x, y, z is the coordinate system of the measurement, following the standard nomenclature of BCDI experiments in which one defines z perpendicular to the top facet and parallel to the scattering vector $\boldsymbol{g}$ of the 111 Bragg peak.

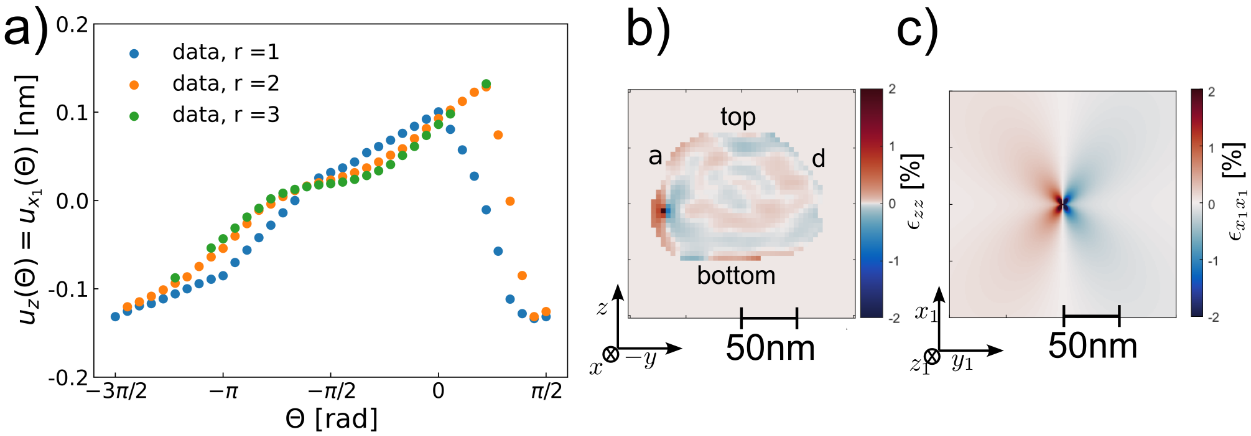

Figure S 13: **Comparison of measured displacement, measured strain and calculated strain.** a) Displacement at different radii r. The numbers denotes the numbers of voxel chosen as radius, one voxel (so the radius of $r = 1$) is 4.17 nm. b) Cut through the reconstructed strain component ϵ_{zz} and c) cut through the calculated strain $\epsilon_{\mathrm{x_1x_1}}$ with $\nu = 0.35$ and $b = 0.388/\sqrt{3}$ nm. Note that the coordinate systems are defined, so that z is parallel to x_1.

References

(1) Kriegner, D.; Wintersbergerb, E.; Stangla, J. xrayutilities: a versatile tool for reciprocal space conversion of scattering data recorded with linear and area detectors. *J. Appl. Crystallogr.* **2013**, *46*, 1162–1170.

(2) Henke, B.; Gullikson, E.; Davis, J. Index of Refraction. 2025-01-22; `https://henke.lbl.gov/optical_constants/getdb2.html`.

(3) Noei, H.; Vonk, V.; Keller, T. F.; Röhlsberger, R.; Stierle, A. DESY NanoLab. *Journal of large-scale research facilities* **2016**, *2*, A76.

(4) Arblaster, J. W. Crystallographic Properties of Platinum. *Platinum Metals Review* **1997**, *41*, 12–21.

(5) Arblaster, J. W. Crystallographic Properties of Rhodium. *Platinum Metals Review* **1997**, *41*, 184–189.

(6) SurfaceNet, Strontium Titanate (SrTiO3). 2024-09-24; https://surfacenet.de/files/kr_Strontium_Titanate.php.

(7) Li, Y.; Jiang, Y.; Chen, M.; Liao, H.; Huang, R.; Zhou, Z.; Tian, N.; Chen, S.; Sun, S. Electrochemically shape-controlled synthesis of trapezohedral platinum nanocrystals with high electrocatalytic activity. *Chem. Commun.* **2012**, *48*, 9531–9533.

(8) Cheula, R.; Maestri, M.; Mpourmpakis, G. Modeling Morphology and Catalytic Activity of Nanoparticle Ensembles Under Reaction Conditions. *ACS Catal.* **2020**, *10*, 6149–6158.

(9) Dupraz, M. et al. Imaging the facet surface strain state of supported multi-faceted Pt nanoparticles during reaction. *Nature Communications* **2022**, *13*, 3003.

(10) Abuin, M.; Kim, Y. Y.; Runge, H.; Kulkarni, S.; Maie, S.; Dzhigaev, D.; Lazarev, S.; Gelisio, L.; Seitz, C.; Richard, M.-I.; Zhou, T.; Vonk, V.; Keller, T. F.; Vartanyants, I. A.; Stierle, A. Coherent X-ray Imaging of CO-Adsorption-Induced Structural Changes in Pt Nanoparticles: Implications for Catalysis. *ACS Appl. Nano Mater.* **2019**, *2*, 4818–4824.

(11) Hermann, K. Balsac (Build and Analyze Lattices, Surfaces, And Clusters). https://www.fhi.mpg.de/1012355/Balsac, 1991-2024; Version: 2024-07-15.

(12) Walton, J. C.; Wincott, P. L.; Fairley, N.; Carrick, A. V. C. *Peak Fitting with CasaXPS: A Casa Pocket Book*; Accolyte Science, 2010.
